# Inference and design of antibody specificity: From experiments to models and back

**DOI:** 10.1371/journal.pcbi.1012522

**Published:** 2024-10-14

**Authors:** Jorge Fernandez-de-Cossio-Diaz, Guido Uguzzoni, Kévin Ricard, Francesca Anselmi, Clément Nizak, Andrea Pagnani, Olivier Rivoire

**Affiliations:** 1 Laboratoire de physique de l’Ecole normale supérieure, CNRS, PSL University, Sorbonne Université, Université Paris-Cité, Paris, France; 2 Italian Institute for Genomic Medicine, IRCCS Candiolo, Candiolo, Italy; 3 Center for Interdisciplinary Research in Biology (CIRB), Collège de France, CNRS, INSERM, Université PSL, Paris, France; 4 Department of Life Sciences and Systems Biology & Molecular Biotechnology Center - MBC, Universita di Torino, Via Nizza, Torino, Italy; 5 Sorbonne Université, CNRS, Institut de Biologie Paris-Seine, Laboratoire Jean Perrin, Paris, France; 6 DISAT, Politecnico di Torino, Corso Duca degli Abruzzi, Torino, Italy; 7 INFN, Sezione di Torino, Torino, Via Pietro Giuria, Torino Italy; 8 Gulliver, CNRS, ESPCI Paris, Université PSL, Paris, France; Weizmann Institute of Science, ISRAEL

## Abstract

Exquisite binding specificity is essential for many protein functions but is difficult to engineer. Many biotechnological or biomedical applications require the discrimination of very similar ligands, which poses the challenge of designing protein sequences with highly specific binding profiles. Experimental methods for generating specific binders rely on *in vitro* selection, which is limited in terms of library size and control over specificity profiles. Additional control was recently demonstrated through high-throughput sequencing and downstream computational analysis. Here we follow such an approach to demonstrate the design of specific antibodies beyond those probed experimentally. We do so in a context where very similar epitopes need to be discriminated, and where these epitopes cannot be experimentally dissociated from other epitopes present in the selection. Our approach involves the identification of different binding modes, each associated with a particular ligand against which the antibodies are either selected or not. Using data from phage display experiments, we show that the model successfully disentangles these modes, even when they are associated with chemically very similar ligands. Additionally, we demonstrate and validate experimentally the computational design of antibodies with customized specificity profiles, either with specific high affinity for a particular target ligand, or with cross-specificity for multiple target ligands. Overall, our results showcase the potential of leveraging a biophysical model learned from selections against multiple ligands to design proteins with tailored specificity, with applications to protein engineering extending beyond the design of antibodies.

## Author summary

A great challenge in protein science is to relate sequences to physical properties, both to predict physical properties from sequences, and to design sequences with desired phenotypes. A promising solution lies in integrating large-scale selection experiments, high-throughput sequencing, and machine learning techniques. However, existing models often focus solely on the property under selection (“fitness”), lacking interpretability. This limits our fundamental understanding of proteins and our ability to engineer them for desired properties, especially those not directly selectable in experiments. Previous studies have shown that incorporating biophysical constraints into models can offer quantitative insights, particularly in transcription factors. Here, we demonstrate that when coupled with extensive experiments, such modeling can not only predict physical features but also design new proteins with specific properties. Our demonstration involves a problem of primary biotechnological and biomedical interest: the design of antibodies with defined specificity profiles. We focus on one of the most challenging tasks in the field, designing antibodies capable of discriminating between structurally and chemically similar ligands. This approach has applications for creating antibodies with both specific and cross-specific binding properties and for mitigating experimental artifacts and biases in selection experiments. The combination of biophysics-informed modeling and extensive selection experiments holds broad applicability beyond antibodies, offering a powerful toolset for designing proteins with desired physical properties.

## Introduction

Proteins often exhibit a delicate balance of multiple physical properties. A prominent example is binding specificity, where some ligand interactions are advantageous while others are detrimental. Examples include transcription factors, which recognize specific DNA motifs among a myriad of alternatives [1], enzymes with a strong preference for their substrate over many similar molecules [2, 3], and immune receptors capable of distinguishing a pathogenic molecule from many others, in particular self molecules [4]. Due to the close chemical similarity between favorable and unfavorable ligands, and/or the dissimilarities between favorable ligands, the engineering of such proteins poses formidable challenges. For instance, in the particular case of therapeutic antibodies, the desired specificity profile typically consists of strong binding affinity to the target antigen while retaining low binding affinity to human self antigens to avoid auto-immune reactions. Additionally, when the target antigen is a human protein, e.g. a tumor marker, antibody cross-specific binding to the human and the cyno and/or murine homologous antigens is often desired to ease drug development [5].

Presently, methods for obtaining specific binders essentially rely on *in vitro* selection experiments [6]. Phage or ribosome display [7, 8] with one immobilized targeted ligand in the presence of soluble non-targeted ligands allows screening for specific binding to the targeted ligand [9]. Yeast display combined with fluorescent-activated cell sorting [10] additionally offers the unique possibility to control precisely specificity selection criteria (including cross-specificity) upfront during the screening process by monitoring fluorescence associated with the targeted and non-targeted ligands in different channels [11], albeit with a maximum library size that is several orders of magnitude smaller.

High-throughput selection can be combined with high-throughput sequencing read-out to identify binders beyond the top hits [12–14], but all experimental approaches are limited by the maximal library size, ranging from typically 10^8^ (yeast), 10^10^ (phage) to 10^15^ (ribosome). As large as these numbers may appear, they represent a negligible fraction of the combinatorially large space of possible sequences. Moreover, experimental screening for specificity requires the targeted and non-targeted ligands to be physically separable, which may be complicated if not impossible in some cases, for instance when considering distinct epitopes on the same molecule. Finally, in experiments, non-targeted ligands are inevitably present, since targeted ligands are typically attached to a cell, a tube/plate, or a magnetic bead.

Recently, works combining high-throughput sequencing and machine learning have demonstrated the possibility of making predictions beyond the scope of experimentally observed sequences [15, 16]. While past works predominantly focused on a single protein property (binding, stability, or catalysis) directly linked to the selection criterion [17], a few studies have shown the feasibility of inferring multiple physical properties, including quantities that are not directly measured [18]. Notable successful examples include predicting thermal stability from binding affinity measurements [19], and inferring specificity profiles of transcription factors from the selective enrichment of DNA sequences [20, 21]. Several recent works have started to apply this type of approach to predict and design antibody specificity [22–25]. Closest to our approach is a recent work showing how a counter-selection to eliminate off-target antibodies, a major difficulty in therapeutic antibody development, can be achieved more efficiently computationally than experimentally [26]. This approach effectively classifies antibody sequences observed in multiple selection experiments to extract those of nonspecific antibodies that bind several (potentially unrelated) targets. The present work addresses a different problem and follows a distinct approach. Our approach is based on a biophysically interpretable model [27] which, besides identifying off-target antibodies from multiple selection experiments, can be applied to disentangle the different contributions to binding to several epitopes from a single experiment. This allows us to address the challenging problem of designing new, experimentally untried antibody sequences that discriminate closely related ligands.

Our biophysics-informed model is trained on a set of experimentally selected antibodies and associates to each potential ligand a distinct binding mode, which enables the prediction and generation of specific variants beyond those observed in the experiments. To showcase this approach, we conducted a series of phage display experiments involving antibody selection against diverse combinations of closely related ligands. First, we demonstrate the model’s predictive power by using data from one ligand combination to predict outcomes for another. Second, we show its generative capabilities by using it to generate antibody variants not present in the initial library that are specific to a given combination of ligands. Our results highlight the potential of biophysics-informed models to identify and disentangle multiple binding modes associated with specific ligands. This approach has applications in designing antibodies with both specific and cross-specific properties and in mitigating experimental artifacts and biases in selection experiments.

## Results

We designed phage display experiments for the selection of antibody libraries and performed two distinct experimental campaigns: in the first, we selected antibodies against various combinations of ligands. This provided us with multiple training and test sets, which we used to build and assess our computational model. In the second, we tested variants predicted by our model but not present in the training set to assess the model’s capacity to propose novel antibody sequences with customized specificity profiles.

The presentation of our results is based on the distinction between the following related terms. We select antibodies for binding to different “complexes”, i.e., different combinations, of “ligands”. The ligands we consider are either small DNA hairpins, or magnetic beads to which these hairpins are attached. A ligand can itself be made up of different epitopes, the parts of the ligand in physical interaction with the antibodies. In the model, the different ways in which an antibody can be selected are inferred through distinct “modes”. These modes may correspond to binding to a complex, a ligand or an epitope, or may more generally describe another thermodynamic state impacting selection. We refrain from using the term “target”, which is used with different meanings in different contexts, either as the complex against which selection is performed, or as the ligand targeted by the experimentalist.

### Experimental selection

Following our previously established protocols [13, 14], we carried out phage-display experiments with a minimal antibody library based on a single naïve human *V*_*H*_ domain in which four consecutive positions of the third complementary determining region (CDR3) are systematically varied to a large fraction of the 20^4^ = 1.6 10^5^ combinations of amino acids (“Germline library” [14]). This library is small enough to allow a high-coverage of its composition by high-throughput sequencing. Out of the 20^4^ potential variants, 48% are observed by sequencing, while we consider the remaining ones to be absent or unobserved. We previously showed that this library of limited size contains antibodies that bind specifically to a diversity of ligands, including proteins, DNA hairpins, and synthetic polymers [13, 14].

**Fig 1:**
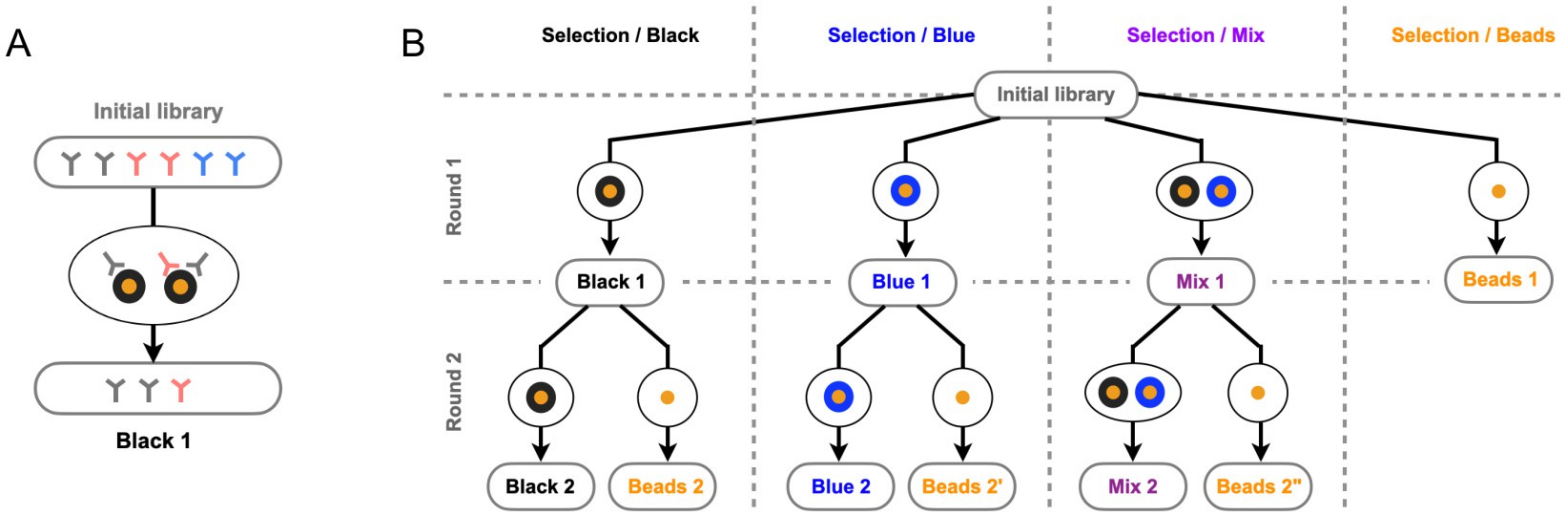
**A**. In a phage display experiment, an initial library containing ∼ 10^5^ variants, each in ∼ 10^6^ copies (here illustrated with 3 variants in 2 copies) is incubated in the presence of DNA hairpins (in black) coupled to magnetic beads (in orange). Antibodies are selected in proportion to their binding probability. The input and output populations are sampled and sequenced to provide data-sets of ∼ 10^5^ sequences each. **B**. We selected the same initial library against four different combinations of ligands: two different DNA hairpins coupled to magnetic beads, presented either alone or in combination, and naked magnetic beads. We refer to these four combinations as “Black”, “Blue”, “Mix” and “Beads” complexes. For the Black, Blue, and Mix complexes, we made two successive rounds of selection. The 10 boxes at the tip of the arrows indicate the 10 sequencing datasets thus produced to feed our model, in addition to the sequencing dataset from the initial library.

Here, we perform selections against complexes comprising two types of ligands, DNA hairpin loops and the surface of streptavidin-coated magnetic beads on which the DNA hairpins are immobilized. We performed independent selections against two such complexes, referred to as “Black” for one DNA hairpin on beads, and “Blue” for another DNA hairpin on beads, as well as selections against mixtures of Black and Blue complexes (“Mix”). Following standard protocols, we performed two rounds of selection with an amplification step in between, where each selection is preceded by an incubation of the phages with naked beads to (partly) deplete the antibody library of bead binders (see Fig A in S1 Text) These pre-selections provided us with data with a fourth selective pressure where naked beads are the only ligand (“Bead”). Importantly, we systematically collected phages at each step of the protocol to closely monitor the antibody library composition. Input phages, phages bound to naked beads during the pre-selection step, and output phages bound to DNA hairpin-coupled beads during the selection step were thus collected followed by infection of *E. coli* bacteria to extract plasmids used as a template for high-throughput sequencing (see Fig A in S1 Text). The relationships between the 8 selection experiments are represented in Fig 1, together with the sequencing data that we collected.

For each experimental selection round *t*, empirical enrichments were computed for each sequence *s* as *ϵ*_*st*_ = *R*_*st*′_/*R*_*st*_, where *R*_*st*_ and *R*_*st*′_, denote respectively the sequencing counts before and after selection. Enrichments against the Black and Blue complexes are observed to be very correlated, consistent with their close chemical similarities (Fig D in S1 Text). Enrichments against one complex and the beads alone are less correlated, indicating both that the beads are not dominant epitopes when coupled to DNA hairpins, and that they are chemically more dissimilar from these hairpins (Fig L in S1 Text).

### A model for multiple-specific selection

We built a computational model where the probability *p*_*st*_ for an antibody sequence *s* to be selected in a particular experiment *t* is expressed in terms of selected and unselected *modes*. Each mode *w* is mathematically described by two quantities: *μ*_*wt*_ that depends only on the experiment *t*, and *E*_*ws*_ that depends on the sequence, such that

$$\begin{array}{c} p_{st}=\frac{\sum_{w\in S_{t}}e^{\mu_{wt}-E_{ws}}}{\sum_{w\in S_{t}}e^{\mu_{wt}-E_{ws}}+\sum_{w\in N_{t}}e^{\mu_{wt}-E_{ws}}}, \end{array}$$ (1)

where $S_{t}$ and $N_{t}$ represent, respectively, sets of selected and not-selected modes available in experiment *t*. Eq (1) is rooted in the thermodynamics of binding at thermal equilibrium [28]: if a molecule can be in one of the selected ($S_{t}$) and unselected ($N_{t}$) thermodynamical states, its probability to be selected is given by (1), which corresponds to a Boltzmann law with *E*_*ws*_ = Δ*F*_*ws*_/*RT*, where Δ*F*_*ws*_ represents the free energy of *s* in state *w*, *R* the universal gas constant and *T* the temperature. A selected state can represent binding to a targeted ligand *w*, in which case *μ*_*wt*_ = ln[*w*], where [*w*] is the relative concentration of free ligand *w* in the experiment *t* (up to a scaling factor). The formula further includes an unselected unbound mode, to account for the possibility that the molecule remains in solution instead of binding any ligand.

Given that our experiments include three types of ligand—two DNA hairpins and magnetic beads—our model comprises four binding modes in total. A bead-bound mode is always selected, the DNA hairpin-bound modes are either selected or absent depending on the ligand present in the experiment, and the unbound mode is always unselected (Fig C in S1 Text). In addition to these physical modes associated with the thermodynamics of binding, our model can incorporate extra pseudo modes not related to binding, to account for biases that may occur during phage production and antibody expression stages (Materials and methods for details). For each mode *w*, *E*_*ws*_ is parametrized by a shallow dense neural network (Materials and methods). During training, the model parameters are optimized globally to capture the evolution of antibody populations across several experiments. Through this optimization process, the initial library abundances are also inferred (Materials and methods). Once the model is trained, it can be used to simulate experiments with a custom set of selected/unselected modes, enabling the prediction of the expected probability of selection of variant reads, which can be compared to empirically observed enrichments of sequence counts in actual experiments.

Furthermore, we verified that introducing more complexity into the model along two different directions had a negligible impact. First, sequences recovered after one round of selection must be amplified before undergoing another round of selection, which occurs through phage infection and may be subject to biases. We collected sequencing data right before and after amplification and verified that no significant amplification bias was present (Fig F in S1 Text). Second, our model considers antibody sequences at the amino-acid level but selection can potentially occur at a nucleotidic level as well. We analyzed the data at this level and confirmed that no significant codon bias was observed in our experiments, consistent with an interpretation of the selection modes as arising primarily from ligand binding (Fig H in S1 Text). We also considered explored other parameterizations of the modes *E*_*ws*_; see Fig M in S1 Text for a comparison justifying our final choice.

### The model disentangles the effect of mixed ligands

To assess the model’s ability to disentangle the contribution to the selection of the different ligands, we conducted two types of validation.

#### Predicting selection against unseen mixtures of ligands

**Fig 2:**
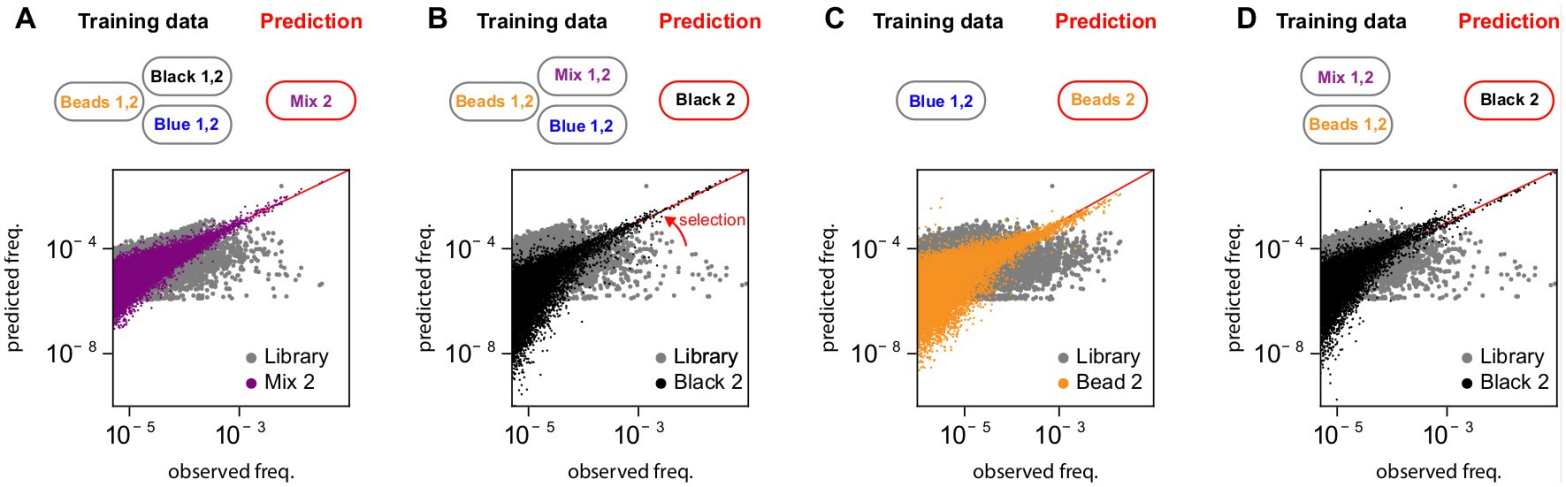
The model predicts accurately the evolution of sequence variants abundances in response to multiple selective pressures. We considered different tasks of increasing difficulty, depending on the training set used: **A.** Model trained on the experiments with Black, Blue complexes, and empty Beads, and prediction evaluated with a mixture of the Black and Blue complexes; **B.** Model trained on experiments with a mixture of Black and Blue complexes, Blue complexes only, and naked Beads, with predictions evaluated on the experiment with Black complexes only; **C.** Model trained on experiments with Blue complexes only, and predictions evaluated on experiments with naked Beads; **D.** Model trained on experiments with a mixture of Black and Blue complexes and naked Beads, and predictions evaluated on experiments with Black complexes only. The panels show scatter plots of the observed (*x*-axis) vs. predicted sequence frequencies (*y*-axis), with the initial library abundances shown in gray for comparison. The Pearson correlation between empirical enrichments and the model-predicted enrichments for each task are given in the legend and in Table B in S1 Text. In all cases p-values (from Student’s test) are < 10^−90^. See SI Mathematical supplement for details about model training.

In the first validation, we trained the model using selection experiments against the Black and Blue complexes consisting of DNA hairpins attached to magnetic beads, and used the inferred model to predict the outcomes of experiments where these two complexes are mixed in equal proportions, which defines the Mix complex (Fig 1). To assess the model’s performance, we compared the read counts of variants in the validation set with the abundances predicted by the model (Fig 2A), and estimated the correlation between predicted probabilities of selection *p*_*st*_ and experimentally determined enrichments *ϵ*_*st*_ against Mix (Table B in S1 Text). The results validate the model’s capacity to integrate different selection experiments to predict the results of selection experiments with unseen combinations and proportions of ligands. As a control, enrichments predicted using only one mode result in significantly lower correlations, confirming that both Black and Blue modes are necessary to model selection in the Mix experiment (Table B in S1 Text).

#### Predicting selection against hidden ligands

In the second validation phase, we trained the model to predict selections against unseen subsets of ligand combinations. We considered three scenarios of increasing complexity: (i) using the data from the Mix, Beads, and Blue selections to disentangle the Black mode and predict the experiment with the Black complex (Fig 2B), (ii) disentangle the effect of Beads using Blue data exclusively and predict the Beads selection (Fig 2C), and (iii) disentangle the Black ligand effect from Mix and Beads selections and predict the Black selection experiment (Fig 2D). The second task is more challenging than the first because the beads in the Blue complex are subdominant epitopes (Fig L in S1 Text), and the third task is more challenging than the other two because the two hairpins are very similar (Fig D in S1 Text) and not seen independently.

As previously, we compared in each case predicted enrichments to empirical enrichments from experiments and obtained very good correlations (Fig 2 and Table B in S1 Text). Altogether, these results validate the model’s capacity to disentangle the contributions of different ligands, and effectively “subtract” the contribution of some ligands to predict the contribution of others.

### Generation and validation of antibodies with custom specificity profiles

In addition to predicting the outcome of experiments involving new combinations of ligands, our model can be employed to design novel antibody sequences with predefined binding profiles. These profiles can be either cross-specific, allowing interaction with several distinct ligands, or specific, enabling interaction with a single ligand while excluding others. The generation of new sequences relies on optimizing over *s* the energy functions *E*_*sw*_ associated with each mode *w* in (1). To obtain cross-specific sequences, we jointly minimize the functions *E*_*sw*_ associated with the desired ligand. On the contrary, to obtain specific sequences, we minimize *E*_*sw*_ associated with the desired ligand *w* and maximize the ones associated with undesired ligands.

**Fig 3:**
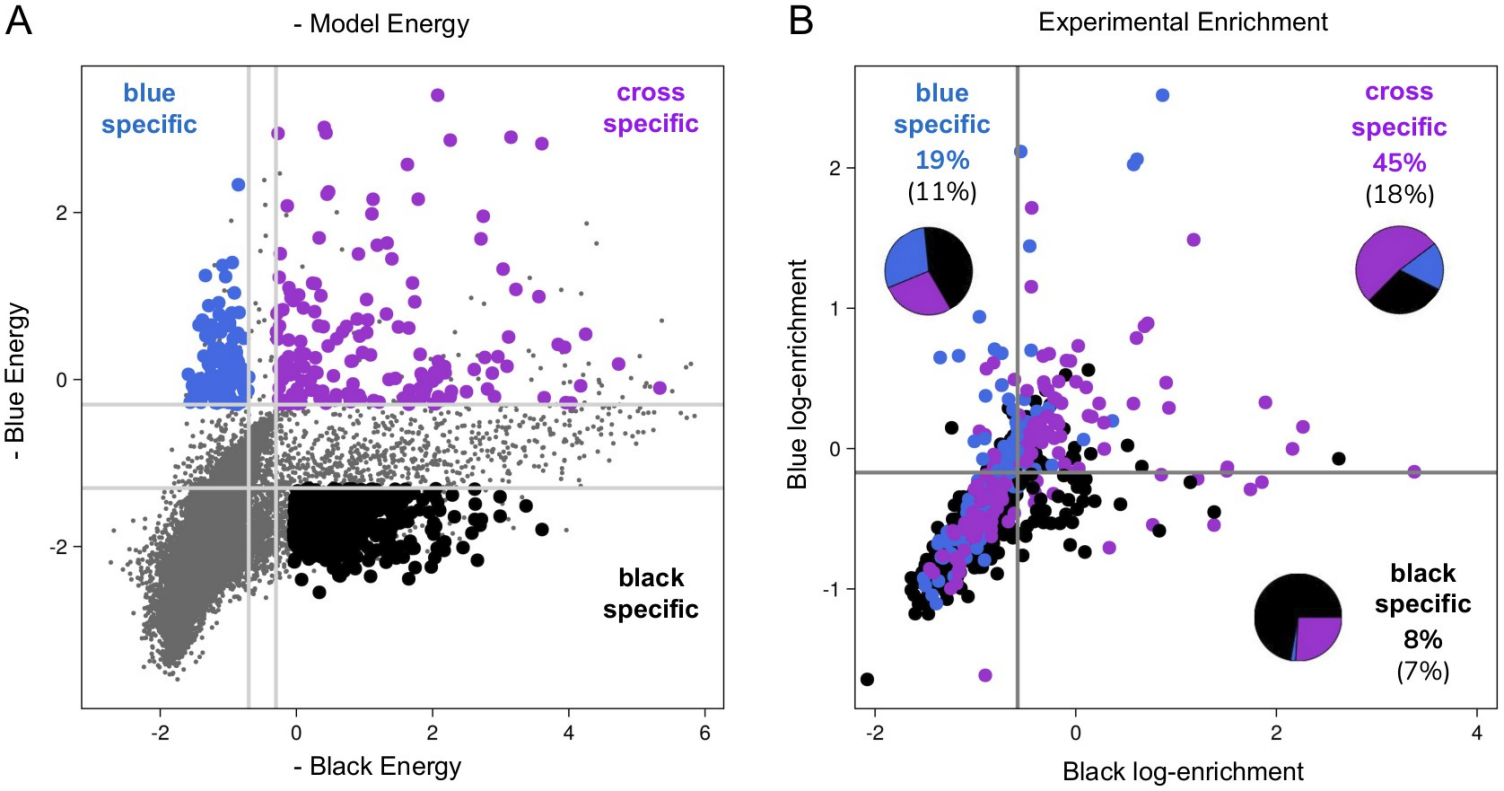
Design and validation of antibodies with prescribed specificity. **A.** Model-based energy plot where each sequence *s* is represented as a circle with coordinates ($E_{sw_{1}},E_{sw_{2}})$, with *w*_1_ representing the binding mode associated with the Black hairpin and *w*_2_ with the Blue hairpin. Sequences predicted to be specific to the Blue hairpin, specific to the Black hairpin, or cross-specific to the two hairpins are respectively highlighted in blue, black, and purple. We selected for experimental validation all the colored sequences that are not present in the training set. **B.** Experiment-based enrichment plot of the selected sequences where each sequence *s* is represented as a circle with coordinates (log *ϵ*_*s*Black_, log *ϵ*_*s*Blue_), with *ϵ*_*s*Black_ representing the enrichment against the Black complex and *ϵ*_*s*Blue_ against the Blue complex. Sequences with high enrichment in one experiment and low enrichment in the other are ligand-specific, those with high enrichment in both are cross-specific, and low-enrichment sequences are non-binders (false positives). We assess our computational approach’s effectiveness by calculating the percentage of designed sequences falling within the correct region. Cross-specific designed antibodies achieve a 45% true positive rate, while Black and Blue-specific binders yield lower percentages (19% and 8%, respectively), reflecting the capacity of our approach to design antibodies with desired properties despite the challenges arising from the close similarity of the two ligands (% in parenthesis indicate the total fraction of sequences in each quadrant; see also Fig I in S1 Text for the choice of the thresholds and Table C in S1 Text for p-values).

Panel A of Fig 3 illustrates the distribution of sequences in the energy plane defined by the modes associated with the two DNA hairpins, as inferred when using all available data. Among all possible sequences, we select those not present in the initial library (thus not included in the training set) and predicted to possess specific binding profiles: sequences in blue are predicted to bind strongly to the Blue DNA hairpin while exhibiting weak binding to the Black DNA hairpin, and reciprocally for those in black, while those in purple are predicted to bind both hairpins.

We validated experimentally these predictions by phage display selection of a library composed of ∼ 2000 computationally designed sequences. Panel B of Fig 3 provides a summary of the results. The enrichments of variants in the two experiments are displayed, with variants above two predefined thresholds considered as binders. These thresholds are taken as the mean values of the two experimental enrichments, a choice that is consistent with the observed enrichment of ∼ 10 control sequences chosen based on their enrichments to be either specific or cross-specific to the Black and Blue complexes (Fig I in S1 Text). We verified that our results are robust to the choice of the thresholds (Fig J in S1 Text).

The four regions represent specific binders for Black and Blue DNA hairpins, cross-specific binders, and non-binding variants. Percentages of the designed antibodies that fall within the respective regions (true positives) are reported, along with the fraction of the total number of points for comparison (with statistical significance reported in Table C in S1 Text). Additionally, the composition of variants within the region segmented by designed specificity is presented. Taken together, these results demonstrate the capacity of the model to propose multiple sequences with desired specificities. Not all designed antibodies have the desired properties, but it must be stressed that the results of Fig 3 address the case of two very similar ligands with the further constraint that the initial library already covers half of the potential diversity, which leaves a relatively small novel design space. In contrast, designing binders to a single ligand regardless of their affinity to the others is comparatively easier (Fig J in S1 Text).

## Discussion

In this study, we propose a multi-stage method that combines high-throughput sequencing of coordinated phage-display experiments, with a machine-learning approach that trains a biophysical model. This model is designed to capture statistical patterns associated with different aspects of the selective pressures to which antibodies are experimentally subjected. By disentangling the different factors influencing selection, we can design sequences with novel combinations of physical properties, making the most of the wealth of information contained in high-throughput sequencing data from selection experiments.

Over the past few years, many machine learning approaches have been applied to the design of antibodies with high affinity and specificity. Many of these approaches require a well-characterized target whose atomic structure is available [29–31]. In contrast, our approach uses sequence data from experimental selection as the only input. This makes it applicable to cases where the ligand is poorly characterized, and in fact provides a means of deducing information about the ligand, through the identification of different binding modes that can be interpreted as different epitopes. Other machine learning approaches have been developed with the aim of analyzing antibody selection experiments to propose new antibody variants with improved binding affinities for a prescribed target, given particular constraints. These constraints include parameters such as viscosity, clearance, solubility, and immunogenicity [23], which are important for drug development, or non-specific binding [24], to eliminate antibodies that tend to bind indiscriminately to a large number of antigens. Some of these works are based on experimental data similar to ours, combining selections against multiple targets with a similar aim of extracting target-specific features, but do not result in biophysically interpretable computational models [22, 26].

In addition, our work differs from these studies in the difficulty of the task we are tackling. We focus indeed on inferring and designing high levels of binding specificity, which involves discriminating between molecular targets with significant structural and chemical similarities. To provide a clear proof of concept, we considered two targets that are not of direct biomedical interest but whose similarity is well characterized. Our two 24-nucleotide hairpins thus differ only by 7 nucleotides in their loop. This difference is comparable to the difference between DNA sequences recognized by transcription factors or restriction enzymes, some of the most specific proteins found naturally. Generating data and developing a model from which to design sequences that discriminate between these two targets is a very stringent test.

A practical application of our approach is the design of new protein candidates with prescribed specificity profiles. The minimal breadth of our initial library reduces the possibility of testing entirely new variants since ≈ 50% of available sequence space is included in the training data, but our approach is also applicable to libraries of greater breadth. As these libraries are necessarily much more undersampled, the potential for discovering better variants is greater, although undersampling can also lead to less accurate models. Characterizing how the potential for discovery and model accuracy scale with library size is a major open question that requires new experiments to be conducted where more sites are randomized, beyond the four sites in the CDR3 that are varied in our libraries. Finally, although not all the variants proposed by our model proved experimentally to have the desired properties, a significant fraction did, which is enough to provide several alternative sequences at the typical scale of ∼100 variants that can be tested experimentally.

There are several avenues for extending the scope of our work. One is to increase the diversity of the initial library, which also allows the incorporation of additional physical modes associated, for example, with thermal stability. Another is to generate and integrate data from experiments in which ligand libraries are selected to bind to one, or several, binders. Beyond practical applications, these extensions have the potential to provide a general approach to deducing the multiple physical properties encoded in protein sequences.

## Materials and methods

### Phage display selection

Phage display selections were performed essentially as in our previous study [14]. Our ‘Germline’ *V*_*H*_ library [14] and the library of designed sequences are both cloned in the pIT2 phagemid vector. M13KO7 (Invitrogen) was used as a helper phage, and TG1 *E. coli* as a host. M280 streptavidin-coated magnetic beads (Dynal) were used for DNA hairpin immobilization. DNA hairpins are single-stranded DNA oligonucleotides biotinylated at their 5’ end (IDT). For the selection against Mix, beads coupled to the Black DNA hairpin were mixed 50–50 with beads coupled to the Blue DNA hairpin.

Phage display experiments included a pre-selection step with naked beads followed by a selection step with DNA hairpin-coupled beads. Specific to the present study, we collected phages at three steps of the selection process (see Fig A in S1 Text), namely (i) input phages, (ii) output phages bound to naked beads during pre-selection, (iii) output phages bound to DNA hairpin-coupled beads during selection. The exact same washing and elution procedures were applied to naked beads and DNA hairpin-coupled beads prior to infection of TG1 cells. Consistent with efficient selection for DNA hairpin-binding, we typically observed a 10 to 100-fold higher phage titer in elutions from beads coupled to DNA hairpins (10^6^ to 10^7^ phages) than from naked beads (10^5^ phages).

### Sequencing read-out

For each phage sample to be sequenced, an amplicon encompassing the 4 randomized CDR3 sites flanked with Illumina adapters bearing sample-specific indices was produced by PCR on DNA purified from TG1 cells following phage infection. The number of PCR cycles was kept as low as possible to avoid distortion due to amplification biases, which we checked specifically.

The ‘Germline’ library selection was sequenced on the Illumina NextSeq 500 platform, producing 76 bp reads. The in-silico designed library selection was sequenced on the Illumina NextSeq 1000 producing 60+60 bp (paired ends) reads.

### Model training

The model is trained by maximizing the likelihood of the observed sequencing read counts of each sequence *s* in an experiment *t*, that we denote by *R*_*st*_, and which are modeled as a multinomial distribution:

$$\begin{array}{c} P\left(\left\{R_{st}\right\}|\left\{N_{st}\right\}\right)\propto \prod_{s}N_{st}^{R_{st}} \end{array}$$ (2)

where *N*_*st*_ is the estimated abundance of this variant in the experiment. The abundances evolve from one experiment *t* to the next *t*′, according to *N*_*st*′_ ∝ *p*_*st*_*N*_*st*_, where *p*_*st*_ are the selectivities in (1). Iterating this relation, we can express *N*_*st*_ as a function of the abundances in the initial library, *N*_*s*0_. Since the *N*_*s*0_ are not directly observed, they are also inferred by maximum likelihood.

An *L*_2_ squared norm regularization is added to penalize large parameter values. Training is substantially accelerated by splitting the sequences into mini-batches. In practice, we form random batches of 128 sequences, which are re-shuffled at each epoch. Further details are given in Supplementary Materials.

### Processing of the data

Sequences containing stop codons are discarded. They are either sequencing errors or can be enriched during amplification since the expression of the antibody is costly for the bacteria. To further reduce sequencing errors, sequences where the flanking constant regions of the CDR3 do not coincide with the designed framework sequence are also filtered out.

## Supporting information

S1 Text Detailed experimental methods. Details of the experimental setup of phage display, the initial library, and the sequencing procedure. **Mathematical supplement.** Detailed description of the biophysical model, the model training, the sequence-to-energy map, the architecture of the feed-forward neural network, and the model selection. **Table A.** Pearson correlations between predicted and empirical enrichments for independent-site model. **Table B.** Pearson correlations between predicted and empirical enrichments for deep model. Same as Table A in S1 Text, for deep model. **Fig A.** Phage display experiment scheme. **Fig B.** The figure illustrates the modes integrated into the model, each associated with distinct states **Fig C.** Training the model: selected set of modes. The selected modes incorporated in the model vary with each specific round. **Fig D.** Comparison of empirical enrichments of each sequence in different experiments. **Fig E.** Pearson correlations between log-enrichmentsx. **Fig F.** Sequencing reads are collected before and after amplification, after the first round of selection is finished and before starting the second round of selections. Scatter of the normalized counts before (x-axis) and after (y-axis) amplification and histograms of the corresponding enrichment ratios. **Fig G.** Same figure as Fig 2 in the main text, employing using model with independent site as described in SI **Fig H.** Each amino-acid sequence can correspond to several nucleotide variants due to codon degeneracy. The plots show a comparison of empirical enrichments for codon-sequences vs. the equivalent amino-acid sequences. **Fig I.** Experimental enrichment of model-designed sequences and controls. **Fig J.** Validation of high-affinity antibodies generated via single-mode optimization. **Fig K.** Distribution of the number of reads of the initial library. **Fig L.** Histograms of binding energies to Black target, Blue target and beads. **Fig M.** Comparison of models for the sequence-to-energy mapping. **Table C.** Reporting p-values of data in Fig 3 using the Fisher exact test. **Fig N.** Test of model predicted frequencies using 95% set of sequences with the lowest counts for training and predicting the 5% sequences with the highest overall abundances.
(PDF)
