# Supplementary material for: Inference and design of antibody specificity: From experiments to models and back
Source: PLoS Comput Biol. 2024 Oct 14;20(10):e1012522. doi: 10.1371/journal.pcbi.1012522 (PMC11501025; doi:10.1371/journal.pcbi.1012522)
Supplement: S1 Text — Details of the experimental setup of phage display, the initial library, and the sequencing procedure. Mathematical supplement. Detailed description of the biophysical model, the model training, the sequence-to-energy map, the architecture of the feed-forward neural network, and the model selection. Table A. Pearson correlations between predicted and empirical enrichments for independent-site model. Table B. Pearson correlations between predicted and empirical enrichments for deep model. Same as Table A in S1 Text, for deep model. Fig A. Phage display experiment scheme. Fig B. The figure illustrates the modes integrated into the model, each associated with distinct states Fig C. Training the model: selected set of modes. The selected modes incorporated in the model vary with each specific round. Fig D. Comparison of empirical enrichments of each sequence in different experiments. Fig E. Pearson correlations between log-enrichmentsx. Fig F. Sequencing reads are collected before and after amplification, after the first round of selection is finished and before starting the second round of selections. Scatter of the normalized counts before (x-axis) and after (y-axis) amplification and histograms of the corresponding enrichment ratios. Fig G. Same figure as Fig 2 in the main text, employing using model with independent site as described in SI Fig H. Each amino-acid sequence can correspond to several nucleotide variants due to codon degeneracy. The plots show a comparison of empirical enrichments for codon-sequences vs. the equivalent amino-acid sequences. Fig I. Experimental enrichment of model-designed sequences and controls. Fig J. Validation of high-affinity antibodies generated via single-mode optimization. Fig K. Distribution of the number of reads of the initial library. Fig L. Histograms of binding energies to Black target, Blue target and beads. Fig M. Comparison of models for the sequence-to-energy mapping. Table C. Reporting p-values of data in Fig 3 using th [file pcbi.1012522.s001.pdf]

Supplementary Information

Inference and design of antibody specificity: from experiments to models and back

J. Fernandez-de-Cossio-Diaz, G. Uguzzoni, K. Ricard, F. Anselmi, C. Nizak, A. Pagnani, O. Rivoire

I. DETAILED EXPERIMENTAL METHODS

Phage display selection

Phage display selections were performed essentially as in our previous study [1] with our 'Germline' V_H library [1] or the library of designed sequences, both cloned in the pIT2 phagemid vector. M13KO7 (Invitrogen) was used as a helper phage, and TG1 *E. coli* (Agilent) as a host. Beads coupled to DNA were prepared by adding 10 μL of 400 μM biotinylated ssDNA target (IDT) incubated for 15mn with 50 μL M280 streptavidin-coated magnetic beads (Dynal) that had been washed prior according to the manufacturer's instructions, followed by two additional washing steps. The Black biotinylated ssDNA target sequence is biotin-AAAAGACCCCATAGCGGTCTGCGT. The Blue biotinylated ssDNA hairpin sequence is biotin-AAAAGACTTGGTAATAGTCTGCGT. Both ssDNA molecules form a hairpin sharing a common stem and bearing different loops. For the selection against Mix, beads coupled to the Black DNA hairpin were mixed 50-50 with beads coupled to the Blue DNA hairpin.

The general scheme of our phage display experiments is described in Fig. S1. Experiments included a pre-selection step with naked beads followed by a selection step with DNA hairpin-coupled beads. Specific to the present study, we collected phages at three steps of the selection process, namely (i) input phages, (ii) output phages bound to naked beads during pre-selection, (iii) output phages bound to DNA hairpin-coupled beads during selection. The exact same washing and elution procedures were applied to naked beads and DNA hairpin-coupled beads prior to phage infection of TG1 cells.

Input phages were produced for 7 h at 30°C following infection with a 20-fold excess of helper phages, and the culture supernatant used as is (no phage precipitation to avoid phage clusters). Our libraries were screened by pre-selection of 10^{11} input phages in 1mL against 50 μL naked magnetic beads for 90 mn, followed by selection of phages that were not bound to naked beads against biotinylated DNA hairpins immobilized on 50 μL magnetic beads for 90 mn. Ten washing steps were performed with 8 mL 0.1% Tween20 (Sigma) prior to elution with 1 mL 100 mM triethylamine (Sigma) for 20mn and neutralization with 0.5 mL Tris 1M pH=7.4 (Sigma), both on naked beads to recover bead binders and on DNA hairpin-coupled beads to recover DNA hairpin binders via infection of an excess of TG1 cells with collected phages. Input phages were also used to infect TG1 cells by adding 500 μL TG1 to 500 μL of a 100-fold dilution of the input phage stock.

Consistent with efficient selection for DNA hairpin-binding, we typically observed a 10 to 100-fold higher phage titer in elutions from beads coupled to DNA hairpins (10^6 to 10^7 phages) than from naked beads (10^5 phages).

Cloning of designed sequences

The library of $\approx$ 2000 designed sequences was constructed by PCR-amplification with the Q5 High-fidelity 2x Master mix (New England Biolabs) of a 120 bp oligo pool (Twist Bioscience), the sequence of which encompasses the 4 randomized CDR3 sites, followed by homology-based assembly (HiFi NEBuilder, New England Biolabs) cloning into the same pIT2 vector as our 'Germline' V_H library.

Sequencing read-out

TG1 cells used for phage infection (input, output from naked beads, output from DNA hairpin-coupled beads), grown on solid plates overnight, were scraped and DNA extracted using a miniprep kit (Macherey-Nagel). A 200bp amplicon encompassing the 4 randomized CDR3 sites flanked with Illumina adapters bearing sample-specific indices was produced in 2 PCR steps. A first PCR uses purified pIT2 plasmid from TG1 cells as a template and staggering oligonucleotides (to favor clustering, as the upstream and downstream flanking sequences of CDR3 sites are constant) to add half of the Illumina adapters without indices. A specific pair of staggering oligonucleotides is used for every subsample to be sequenced (input, output with hairpin or without hairpin). A second PCR uses the product of the

first PCR step as template and adds the indices and the remaining part of Illumina adapters. Both PCR steps were carried out with the Q5 High-fidelity 2x Master mix (New England Biolabs) for 15 cycles to avoid distortion due to amplification biases, which we checked specifically.

The 'Germline' library selection was sequenced on the Illumina NextSeq 500 platform, producing 76 bp reads. The in-silico designed library selection was sequenced on the Illumina NextSeq 1000 producing 60+60 bp (paired ends) reads.

Analysis of empirical enrichments

For each experimental selection t, empirical enrichments were computed for each sequence s as

$$\epsilon_{st} = R_{st'}/R_{st} \tag{S1}$$

where R_{st} and $R_{st'}$, denote respectively the sequencing counts before and after selection. Since the empirical estimate Eq. (S1) is susceptible to sampling noise, and can be undefined if R_{st} vanishes, ϵ_{st} is computed only for sequences s for which both $R_{st'}$ and R_{st} are larger than a minimum threshold count. As the diversity of the population decreases between the first and second rounds of selection, it is expected that these enrichments are best estimated at the second round of selection, although for a smaller set of sequences that are more represented.

Fig. S4 compares the enrichments obtained in different experiments, where selection corresponds to binding different targets. Fig. S5 plots the Pearson correlations between these enrichments, as a function of the minimum count threshold imposed in the numerator and denominator of Eq. (S1). Empirical enrichment against the Blue, Black, and Mix complexes show significant correlations (Fig. S5), consistent with the structural and chemical similarity of the two DNA hairpins. This feature reflects our choice to study closely related ligands that are challenging to differentiate. In contrast, empirical enrichments from selection against the Beads are appreciably more distinct from the other ones (bottom rows of Figs S4 and S5).

For variants with more than 50 counts before and after selection, we compute their empirical enrichment and compared this to the model predicted selectivity. Results are shown in Table A. We did this for each of the computational predictions in Fig. 2, and report here the resulting correlations in each case. As a control the last column of Table A reports the correlation if the mode Blue is used to predict the enrichments, instead of the correct one.

II. MATHEMATICAL SUPPLEMENT

Biophysical model of selection

Let N_{st} be the total number of phages carrying sequence s in a library at round t, before selection. In one experimental round, phages are selected by some phenotypic criteria (e.g. binding to a particular ligand). In our model, we consider each sequence to be in one of several possible thermodynamic states w (bound to a particular ligand, in solution, etc.). Each thermodynamic state w is populated by a number of particles n_{swt}, and we have $\sum_w n_{swt} = N_{st}$. Also, each thermodynamic state is described by a sequence-dependent energy function E_{sw}, related to the propensity of a sequence to be found in this state (in the following we describe some possible parametrization of this function). We denote by μ_{wt} the chemical potentials for each state at a particular round, we can then model the abundances n_{swt} as follows,

$$\frac{n_{swt}}{N_{st}} = \frac{e^{\mu_{wt}-E_{sw}}}{\sum_{w'} e^{\mu_{w't}-E_{sw'}}} \tag{S2}$$

In the case of a thermodynamic state corresponding to binding a ligand, the chemical potentials μ_{wt} are proportional to the logarithmic concentration of the available ligand to bind. Next, we define the selectivity of a sequence s in the experimental round t, as

$$p_{st} = \frac{\sum_{w\in\mathcal{S}_t} e^{\mu_{wt}-E_{sw}}}{\sum_{w\in\mathcal{S}_t} e^{\mu_{wt}-E_{sw}} + \sum_{w\in\mathcal{N}_t} e^{\mu_{wt}-E_{sw}}} \tag{S3}$$

Here, we consider a subset of states $\mathcal{S}_t$ which are selected in the current experiment (e.g. bound to ligand), and a set of states $\mathcal{N}_t$ which are depleted (e.g., washed in solution). Together, $\mathcal{S}_t \cup \mathcal{N}_t$ defines the set of feasible thermodynamic states in the experimental conditions of round t. Particles that adopt states $w \in \mathcal{S}_t$, are selected and result in an enrichment of the corresponding sequence. The remaining $(1-p_{st})N_{st}$ particles of sequence s are washed away.

Amplification

After the selection step, there is an amplification step when the overall population size is restored. Assuming that the abundances are normalized, $\sum_s N_{st} = 1$ and denoting by $N_{s,t+1}$ the phage abundances prepared for the next round, we have:

$$N_{s,t+1} = \frac{p_{st} N_{st}}{z_t} \tag{S4}$$

where

$$z_t = \sum_\sigma p_{\sigma t} N_{\sigma t} \tag{S5}$$

is the population size immediately after selection. To restore the original population size, an amplification step is necessary, where the population is multiplied by the amplification factor $1/z_t$.

Multiple rounds

When dealing with multiple selection experiments, the output of a selection round can be used as the input of another selection round. We consider the selection experiments to be arranged into a rooted tree, such as the one depicted in Fig. S3. The nodes represent phage populations at a specific time with the root representing the initial sequence library. The edges represent a selection and amplification process that modifies the population in the parent node to the descendant node. In each branch, the node's generations are denoted by t, with $t = 0$ being the root. In particular, N_{s0} represents the initial library abundances. The parent of a non-root node, $t > 0$, is denoted by $\mathfrak{a}(t)$. Starting from a node t, we can traverse back to its ancestors until we reach the root of the tree. We define by

$$\mathcal{A}(t) = \{t, \mathfrak{a}(t), \mathfrak{a}(\mathfrak{a}(t)), \dots, 0\} \tag{S6}$$

the set of ancestors encountered during this traversal (note that $\mathcal{A}(t)$ includes t itself, for convenience). In particular, for the root node $\mathcal{A}(0) = \{0\}$. For $t > 0$, we have that p_{st} denotes the selectivity of sequence s, in the round of selection that brings the population from $\mathfrak{a}(t)$ to node t. It follows that we can write:

$$N_{st} = \frac{p_{st} N_{s\mathfrak{a}(t)}}{\sum_\sigma p_{\sigma t} N_{\sigma \mathfrak{a}(t)}} = \frac{N_{s0} P_s^t}{Z_t} \tag{S7}$$

for $t > 0$, by induction, where $P_s^t = \prod_{\tau \in \mathcal{A}(t)} p_{s\tau}$ and $Z_t = \sum_s N_{s0} P_s^t$. For the root nodes it is convenient to set $p_{s0} = 1$, $P_{s0} = 1$, $Z_0 = 1$, and $\mathfrak{a}(0) = 0$. Then these formulas remain valid at the root. Given the selectivities p_{st} and the initial abundances N_{s0}, we can use these expressions to determine all future populations of the tree.

Training the model

The data are the counts of sequence reads taken at each sequenced round (that could be a subset of all nodes in the experiment tree), $\{R_{st}\}$. As the result of a sampling and sequencing procedure, the counts are related to the actual abundances by a multinomial distribution:

$$\mathcal{P}(\{R_{st}\}|\{N_{st}\}) = \frac{(\sum_s R_{st})!}{\prod_s R_{st}!} \prod_s N_{st}^{R_{st}} \tag{S8}$$

The abundances of all populations $\{N_{st}\}$ are determined by the initial abundances $\{N_{s0}\}$ and the selectivities, $\{p_{st}\}$. Therefore, we can write $\mathcal{P}(\{R_{st}\}|\{N_{st}\}) = \mathcal{P}(\{R_{st}\}|\{p_{st}\}, \{N_{s0}\})$. Since the initial abundances $\{N_{s0}\}$ are usually unknown, we can train our model by maximizing $\mathcal{P}(\{R_{st}\}|\{p_{st}\}, \{N_{s0}\})$ with respect to the parameters determining the selectivities $\{p_{st}\}$ (to be specified below) and the initial abundances $\{N_{s0}\}$, subject to $N_{s0} \geq 0$ and $\sum_s N_{s0} = 1$. To carry out the maximization over $\{N_{s0}\}$ we can introduce a Lagrangian,

$$\mathcal{L} = \ln \mathcal{P}(\{R_{st}\}|\{N_{s0}\}, \{p_{st}\}) + \lambda \sum_s N_{s0} \tag{S9}$$

where we used the Lagrange multiplier λ for the constraint $\sum_s N_{s0} = 1$. Differentiating with respect to N_{s0} and setting the derivative to zero, gives the equation:

$$\sum_\tau R_{s\tau} - \sum_\tau \frac{R_\tau}{Z_\tau} N_{s0} P_s^\tau + \lambda N_{s0} = 0 \tag{S10}$$

where $R_\tau = \sum_\sigma R_{\sigma\tau}$, and R_τ / Z_τ is the sampling coverage at round τ. Note that:

$$\sum_s N_{s0} \frac{\partial \mathcal{L}}{\partial N_{s0}} = \sum_s N_{s0} = \lambda \tag{S11}$$

Therefore the stationarity conditions $\frac{\partial \mathcal{L}}{\partial N_{s0}} = 0$ imply $\lambda = 0$. This is also intuitively expected, because $\mathcal{L}$ depends only on the relative ratios among the N_{s0}, and would be insensitive to a global increase of the total $\sum_s N_{s0}$ while preserving those ratios. Therefore $\mathcal{L}$ has no gradient orthogonal to the constraint $\sum_s N_{s0} = 1$, making the Lagrange multiplier unnecessary. Now solving for N_{s0}, we obtain:

$$N_{s0} = \frac{\sum_\tau R_{s\tau}}{\sum_\tau P_s^\tau (R_\tau / Z_\tau)} \tag{S12}$$

which gives N_{s0} as functions of Z_t and the selectivities p_{st}. In particular note that $N_{s0} = 0$ for unobserved sequences (those for which $R_{st} = 0$ for all t in the tree). There are two contributions to the initial abundances: the different sampling coverage at different rounds and the effect of selection. In absence of selection, the formula above becomes more intuitive. Then P_{st}/Z_t is a constant independent of s, t, which must be one by normalization. Then, $N_{s0} = \sum_\tau R_{s\tau} / \sum_{\sigma\tau} R_{\sigma\tau}$ is also independent of t, and we just aggregate all the read samples to make an inference of the underlying abundances. The factors $P_{s\tau} R_\tau / Z_\tau$ in the denominator of Eq. (S12), then serve to account for the effect of selection.

By using Eq. (S12) we can consider Z_t as free parameters, in place of the initial abundances N_{s0}. Obtaining the following learning gradient:

$$\frac{\partial \mathcal{L}}{\partial Z_t} = \frac{R_t}{Z_t} \left(\sum_s N_{st} - 1 \right) \tag{S13}$$

Notice that $\mathcal{L}$ as a function of $\{P_s^t, Z_t\}$ depends only on the ratios $Z_t / Z_{t'}$ and is invariant to a multiplication of all the Z_t by a common factor. Therefore we can set $Z_0 = 1$ to break this degeneracy, consistent with the previous definitions. The stationary conditions $\frac{\partial \mathcal{L}}{\partial Z_t} = 0$ reproduce the normalization constraints $\sum_s N_{st} = 1$. It follows that if we treat the Z_t as free parameters, at a stationary point of $\mathcal{L}$ these constraints will be satisfied automatically.

Below, we use $\{p_{st}, z_t\}$ as free parameters in place of $\{P_s^t, Z_t\}$.

Sequence to energy mapping

The selectivities p_{st} are given by Equation (S3). In turn, a mapping giving the energies E_{sw} for each sequence must be parameterized. We considered two models. In the simplest case, all the sites of the sequence are independent, which leads to an additive model,

$$E_{sw}^{\mathrm{IS}} = -\sum_{i=1}^{L} h_{wi}(s_i) \tag{S14}$$

that we call the independent-site model, and where the local fields h_{wi} are learned during model training. This assumption fails to consider epistatic effects between pairs of sites within a mode. To account for these, a possibility is to introduce a Potts-like model with two-body interactions, as typically done in DCA-like approaches [2, 3]. However this results in a large number of coupling parameters ($\sim 20^2 L^2$). More generally, we can consider any functions $E_{sw} = E_w(s)$ assigning energies to sequences in different thermodynamic states. We here considered a feed-forward neural network, taking as input a one-hot encoded sequence, and producing as output the energy, $E_{sw}^{\mathrm{NN}} = E_w^{\mathrm{NN}}(s)$. The parameters of the neural network (NN) are learned during model training. Epistatic interactions arise as non-trivial correlations induced by the non-linearities of the network. We report the details of our architectural choices below.

Low-selectivity regime (or rare binding approximation)

In typical experimental realizations, a very small fraction of variants is selected for the next round. A further simplification can be obtained by exploiting this a low-selectivity regime, where $p_{st} \ll 1$ for all sequences in all rounds. We can then approximate:

$$p_{st} \approx \frac{p_{st}}{1 - p_{st}} = \frac{\sum_{w \in \mathcal{S}_t} e^{\mu_{wt} - E_{sw}}}{\sum_{w \in \mathcal{N}_t} e^{\mu_{wt} - E_{sw}}} \tag{S15}$$

We call this the rare binding approximation [4].

Parametric invariance in the rare-binding approximation

In the low-selectivity regime, a parametric degeneracy arises. Suppose we modify the potentials μ_{wt} as follows:

$$\mu'_{wt} = \begin{cases} \mu_{wt} + a_t & w \in \mathcal{S}_t \\ \mu_{wt} + b_t & w \in \mathcal{N}_t \end{cases} \tag{S16}$$

and

$$\ln z'_t = \ln z_t + a_t - b_t \tag{S17}$$

where a_t, b_t are arbitrary. According to Eq. (S4), the abundances evolve according to the ratios p_{st}/z_t. Under the rare-binding regime,

$$\frac{p'_{st}}{z'_t} = \frac{\sum_{w \in \mathcal{S}_t} e^{\mu_{wt} + a_t - E_{sw}}}{\sum_{w \in \mathcal{N}_t} e^{\mu_{wt} + b_t - E_{sw}}} e^{-\ln z_t - a_t + b_t} = \frac{p_{st}}{z_t} \tag{S18}$$

which shows that p_{st}/z_t remains invariant under the transformations Eqs. (S16), (S17). In other words, the amplification factors become indistinguishable from the chemical potentials. The observed data, which reflects the abundances, cannot separate these two parameters. We must then forego the inference of the amplification factors.

We are free to impose a gauge $\sum_w \mu_{wt} = 0$. This means that a_t, b_t are not completely free, but rather they must satisfy $|\mathcal{S}_t| a_t + |\mathcal{N}_t| b_t = 0$. The remaining degree of freedom can be exploited to enforce $\sum_{w \in \mathcal{S}_t} \mu_{wt} = \sum_{w \in \mathcal{N}_t} \mu_{wt} = 0$, which is stronger than the condition $\sum_w \mu_{wt} = 0$ from the previous section. More precisely, given chemical potentials μ_{wt}, we can choose:

$$a_t = -\frac{1}{|\mathcal{S}_t|} \sum_{w \in \mathcal{S}_t} \mu_{wt}, \tag{S19}$$

$$b_t = -\frac{1}{|\mathcal{N}_t|} \sum_{w \in \mathcal{N}_t} \mu_{wt} \tag{S20}$$

which satisfy $|\mathcal{S}_t| a_t + |\mathcal{N}_t| b_t = 0$, and for which:

$$\sum_{w \in \mathcal{S}_t} \mu'_{wt} = \sum_{w \in \mathcal{S}_t} \mu_{wt} + |\mathcal{S}_t| a_t = 0, \tag{S21}$$

$$\sum_{w \in \mathcal{N}_t} \mu'_{wt} = \sum_{w \in \mathcal{N}_t} \mu_{wt} + |\mathcal{N}_t| b_t = 0 \tag{S22}$$

while the amplification factors transform as:

$$\ln z'_t = \ln z_t - \frac{1}{|\mathcal{S}_t|} \sum_{w \in \mathcal{S}_t} \mu_{wt} + \frac{1}{|\mathcal{N}_t|} \sum_{w \in \mathcal{N}_t} \mu_{wt} \tag{S23}$$

When the chemical potentials satisfy $\sum_{w \in \mathcal{S}_t} \mu_{wt} = \sum_{w \in \mathcal{N}_t} \mu_{wt} = 0$, we will say that we are in the rare binding gauge.

This change of variables shows how one can absorb the amplification factors into the chemical potentials. Alternatively, we can exploit this new gauge invariance to impose that $\zeta_t = 0$, by choosing $a_t = -\frac{|\mathcal{N}_t| \zeta_t}{|\mathcal{S}_t| + |\mathcal{N}_t|}$ and $b_t = \frac{|\mathcal{S}_t| \zeta_t}{|\mathcal{S}_t| + |\mathcal{N}_t|}$.

Architecture of feed-forward neural network

For each state considered, the architecture consists of 3 layers, with 20 and 5 hidden units, with a SeLU nonlinearity [5] in each.

Regularization

For the independent-site model, the regularization penalty has the form:

$$\lambda_{L2} \sum_{ai} h_i(a)^2 \tag{S24}$$

while for the neural network model the sum extends over all weight parameters in the network.

Model training

All models were trained using the AdaBelief [6] optimizer, as implemented in the Flux.jl package of Julia [7]. Models were trained with increasing mini-batch sizes (200, 400, 800, 1000, 1500, 2000, 2500, and 4000), with each mini-batch size kept constant for 700 epochs.

The models used in Figure 2 of the main text, were initialized using the independent models (see Fig. S7), by first training them to minimize the L2 norm of differences between energies assigned to the sequences in our dataset.

Model selection

In this paper we consider a neural network (NN) model to assign state energies to sequence variants. One advantage of deep neural networks over Potts models is that they can have less parameters. In the 3-layer NN we have used in this work, with 20 and 5 hidden units, the number of weights is 1700, while a Potts model has $q^2L(L-1)/2 = 3040$ coupling parameters. NNs become increasingly advantageous for larger proteins. We also found that training of the neural network model was faster, possibly thanks to optimized deep learning libraries used [7, 8]. Another advantage of NN is that they can consider many-body interactions, whereas the standard Potts model is limited to two-body interactions only. For these reasons we were interested in exploring NNs for this paper.

We have conducted a training/validation test to demonstrate the advantage of our NN architecture. We trained three model variants:

- **Indep.**, where the energy assigned to each sequence variant in each thermodynamic state is computed under an independent-site assumption. This model considers only qL site parameters, for each state.
- **Potts**, where in addition to the site fields, the model includes $q^2L(L-1)/2$ pairwise couplings between all pairs of sites.
- **Deep**, where the energy is computed using the NN architecture we have described above.

These models were trained on a randomly selected subset of 80% of the sequences in the experimental data. After training converged, we computed the likelihood of the observed sequence counts, both in the training split, and in the remaining 20% of sequences in a withheld validation split. The results are shown in Fig. S13.

We conclude that the NN outperforms the other two models. In particular, the NN is better than the Potts model, even in the train set, in spite of the fact that the Potts model has more parameters. As explained above, this is likely a consequence of the fact that the Potts model is unable to consider many-body interactions.

The coefficient λ_{L2} from Eq. (S24) was varied over a range between 10^{-4} and 10. The optimal choice in terms log-likelihood of a withheld dataset was chosen, resulting in $\lambda_{L2} = 0.01$.

As a final validation of the model generalization capability, we trained on the 95% subset of sequences with lowest counts across the full experiment, and then tested the predictions on the remaining 5% of sequences. The results are

shown in Fig. 14.

1. S. Schulz, S. Boyer, M. Smerlak, S. Cocco, R. Monasson, C. Nizak, and O. Rivoire, PLoS computational biology **17**, e1008751 (2021).
2. F. Morcos, A. Pagnani, B. Lunt, A. Bertolino, D. S. Marks, C. Sander, R. Zecchina, J. N. Onuchic, T. Hwa, and M. Weigt, Proceedings of the National Academy of Sciences **108**, E1293 (2011).
3. S. Cocco, C. Feinauer, M. Figliuzzi, R. Monasson, and M. Weigt, Reports on Progress in Physics **81**, 032601 (2018).
4. J. Fernandez-de Cossio-Diaz, G. Uguzzoni, and A. Pagnani, Molecular biology and evolution **38**, 318 (2021).
5. G. Klambauer, T. Unterthiner, A. Mayr, and S. Hochreiter, Advances in neural information processing systems **30** (2017).
6. J. Zhuang, T. Tang, Y. Ding, S. C. Tatikonda, N. Dvornek, X. Papademetris, and J. Duncan, Advances in neural information processing systems **33**, 18795 (2020).
7. M. Innes, E. Saba, K. Fischer, D. Gandhi, M. C. Rudilosso, N. M. Joy, T. Karmali, A. Pal, and V. Shah, CoRR **abs/1811.01457** (2018), 1811.01457, URL `https://arxiv.org/abs/1811.01457`.
8. M. Innes, Journal of Open Source Software (2018).

Training data	Prediction	Pearson correlation	Control (Blue)
Beads (1,2), Black (1,2), Blue (1,2)	Mix (2)	0.63	0.61
Beads (1,2), Blue (1,2), Mix (1,2)	Black (2)	0.70	0.54
Blue (1,2)	Beads (2)	0.54	0.12
Mix (1,2), Beads (1,2)	Black (2)	0.71	0.46

TABLE A: Pearson correlations between predicted and empirical enrichments for independent-site model. A model trained on the data from the first column is used to predict enrichments of the experiment in the second column. In these experiments, a simple independent site model (Eq. (S14)) was used. The correlations between empirical log-enrichments and model log-enrichments predictions are given in the third column. To demonstrate that the inferred modes have a meaningful correspondence to the physical modes, the last column shows (as a control) how the correlation decreases if only the Blue mode is used to predict the enrichments. See also Fig. S7. In all cases p-values (from Student's test) are $< 10^{-90}$. Note that the more different the ligands, the easier their discrimination. The fact that training the model solely on the Blue complex and using only the Blue mode leads to the worst correlation can be understood from the observation that, of all the examples of this Table, they are the most similar pair.

Training data	Prediction	Pearson correlation	Control (Blue)
Beads (1,2), Black (1,2), Blue (1,2)	Mix (2)	0.81	0.76
Beads (1,2), Blue (1,2), Mix (1,2)	Black (2)	0.86	0.75
Blue (1,2)	Beads (2)	0.64	0.43
Mix (1,2), Beads (1,2)	Black (2)	0.82	0.58

TABLE B: Pearson correlations between predicted and empirical enrichments for deep model. Same as Table A, for deep model. See also Fig. 3 in the main text.

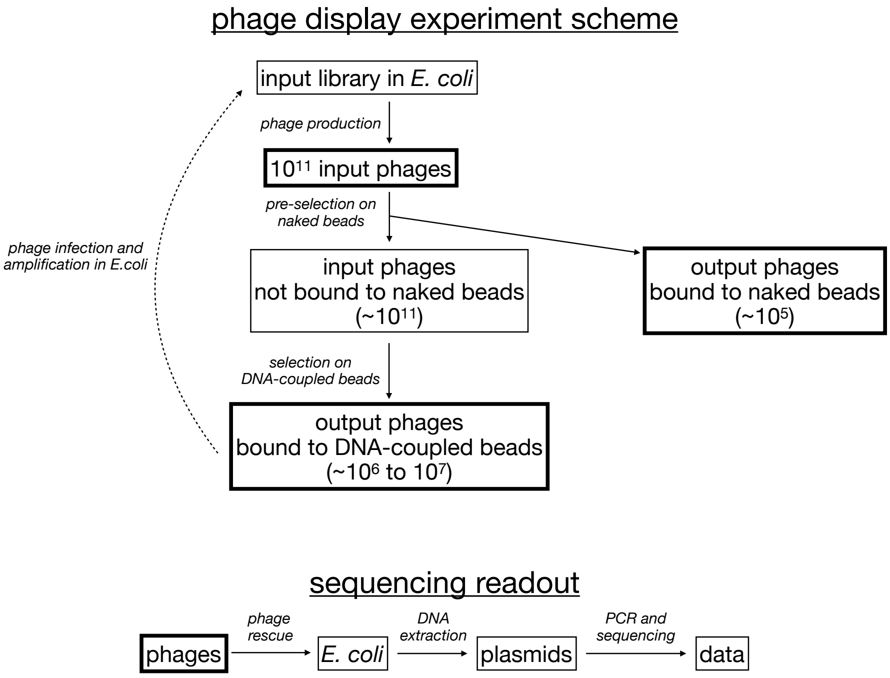

FIG. A: **Phage display experiment scheme.** Input phages are produced from *E. coli* bacteria and first pre-selected against naked beads. Phages that did not bind naked beads are then selected for binding to DNA target-coupled beads. Phages bound to DNA-coupled beads are collected followed by infection of *E. coli* bacteria for the next cycle. **Sequencing readout.** Three separate *E. coli* bacterial cultures are infected with (i) input phages, (ii) output phages bound to naked beads and (iii) output phages bound to DNA-coupled beads. Plasmid DNA is extracted from bacteria to serve as a template for high-throughput sequencing of a PCR amplicon encompassing the 4 randomized antibody CDR3 sites.

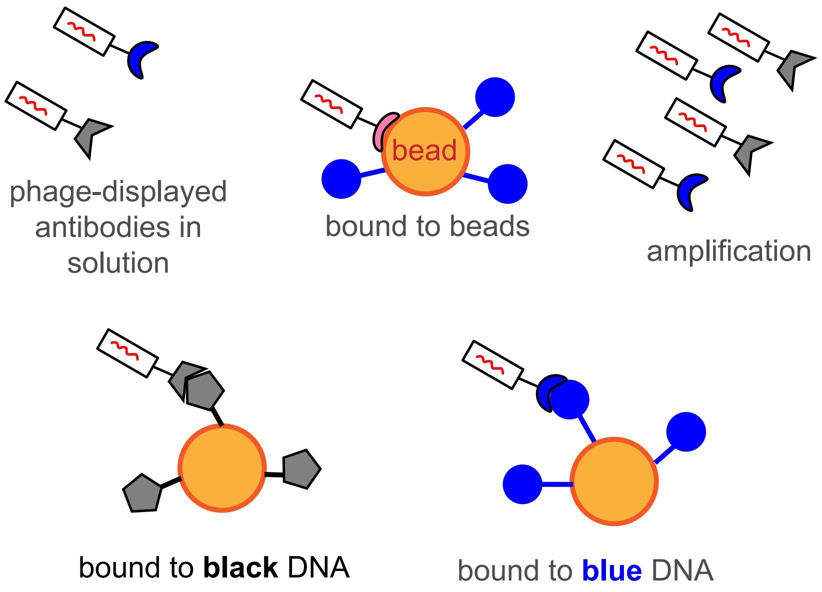

FIG. B: The figure illustrates the modes integrated into the model, each associated with distinct states: The first mode corresponds to an unselected, unbound state, and is a common element in every selection round. The other modes are linked to the binding of either the Black and Blue ligand or the Beads, where they are immobilized. An additional mode is defined in the model, which is not directly related to the physical binding to specific ligands but accounts for the process of amplification and the potential biases that it introduces. The selection or exclusion of these modes depends on the specific selection round, as visually depicted in Fig. S3.

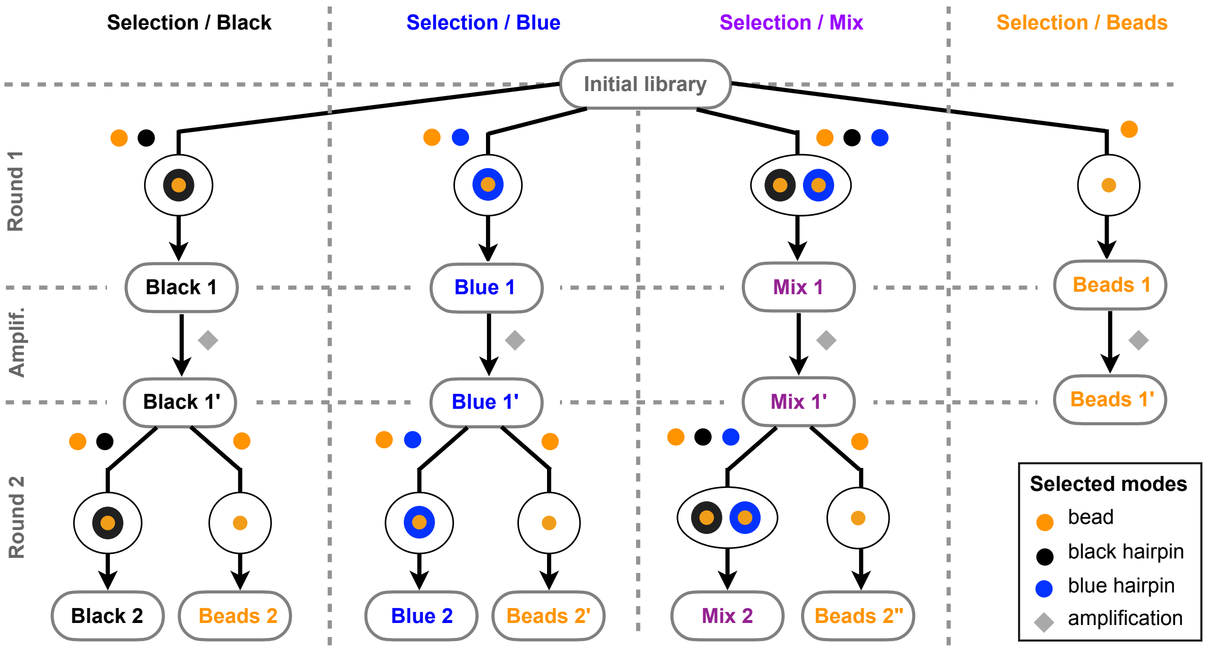

FIG. C: Training the model: selected set of modes. The selected modes incorporated in the model vary with each specific round. This adaptability enables comprehensive training using all available data. In the figure, the tree structure of the experiment (as presented in Figure 1 of the main text) is reported with the annotations of the selected mode for each branch or selection round. The four modes model distinct physical processes depicted in Fig. S2: binding the black hairpin, binding the blue hairpin, binding the supporting bead, and the bias introduced during the amplification process.

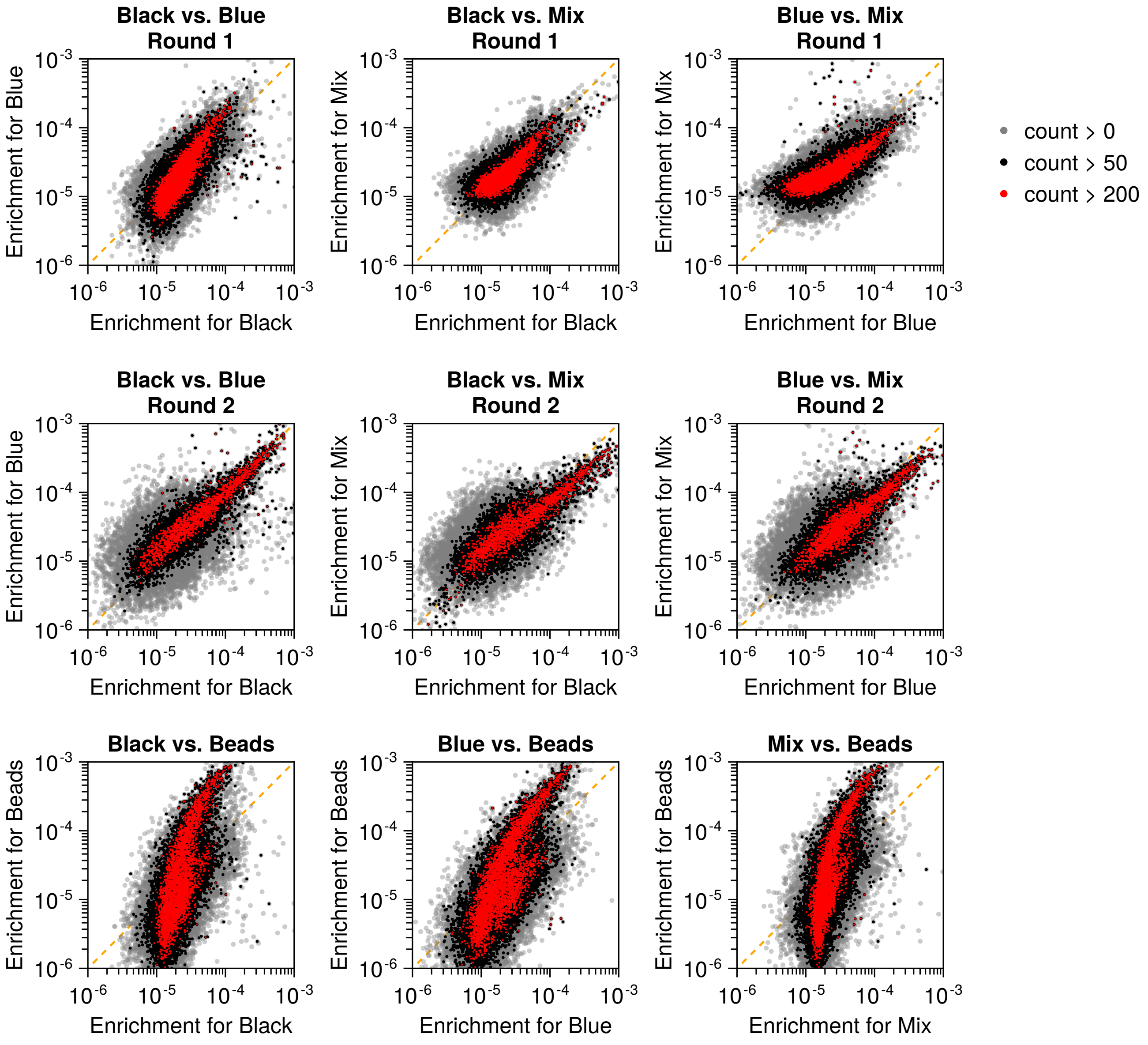

FIG. D: Comparison of empirical enrichments of each sequence in different experiments. The first and second row compare enrichments in the first and second rounds of selection (first and second row), against the Black, Blue, and Mix complexes. In each panel, the x-axis is the enrichment against a ligand (Black, Blue, or Mix), and the y-axis is the enrichment of the same sequence against a different ligand. The bottom row compares the enrichments against the Beads vs. the enrichments against the Black, Blue, and Mix ligands. See Eq. (S1) for the definition of enrichment. Sequences with counts lower than a given threshold are filtered out, as indicated in the legend at the top-right: all sequences with at least one read before and after selection are shown in gray, while sequences with more than 50 (200) reads before and after selection are shown in black (red). The Pearson correlations corresponding to each panel are shown in Fig. S5.

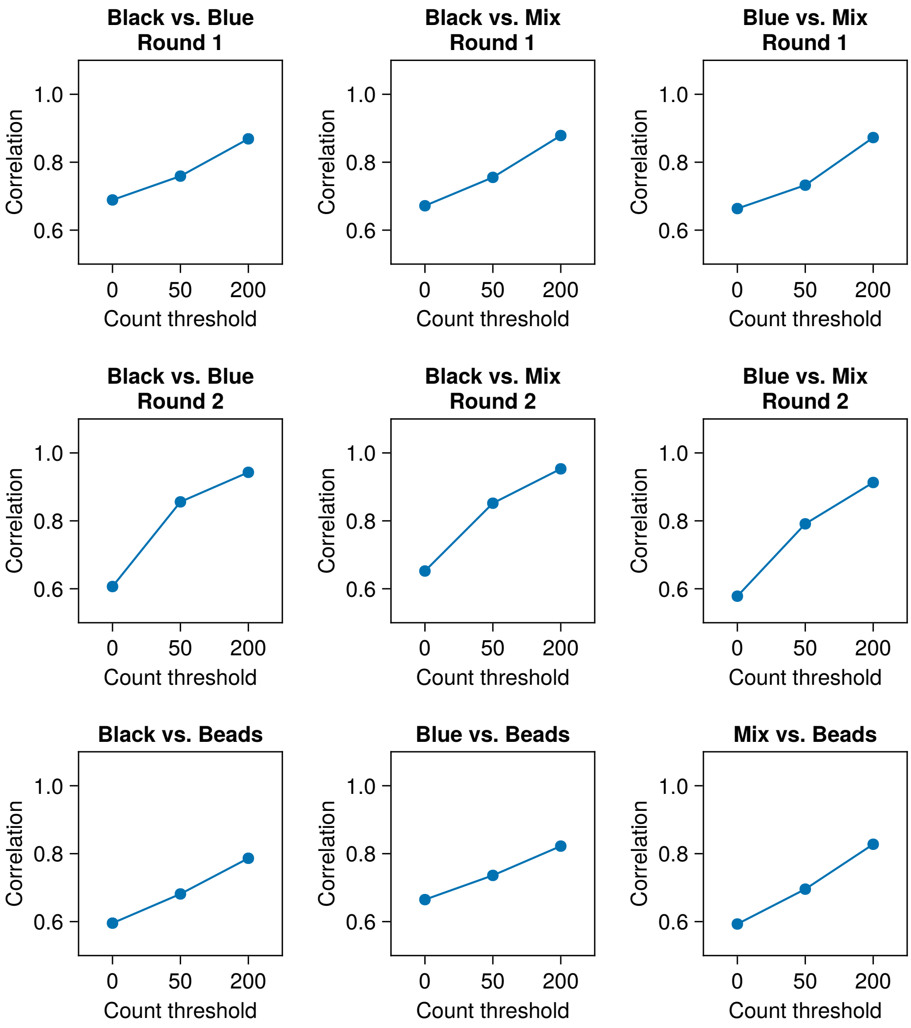

FIG. E: Pearson correlations between log-enrichments ($\log(\epsilon_{st})$, where ϵ_{st} is defined in Eq. (S1)) in different experiments. Each panel shows the correlations between $\log(\epsilon_{st})$ and $\log(\epsilon_{st'})$ for different experiments t, t', indicated in the panel title. The correlations are computed after filtering out sequences with counts lower than a given threshold (indicated in the x-axis) before and after selection. Panels are in correspondence to Fig. S4.

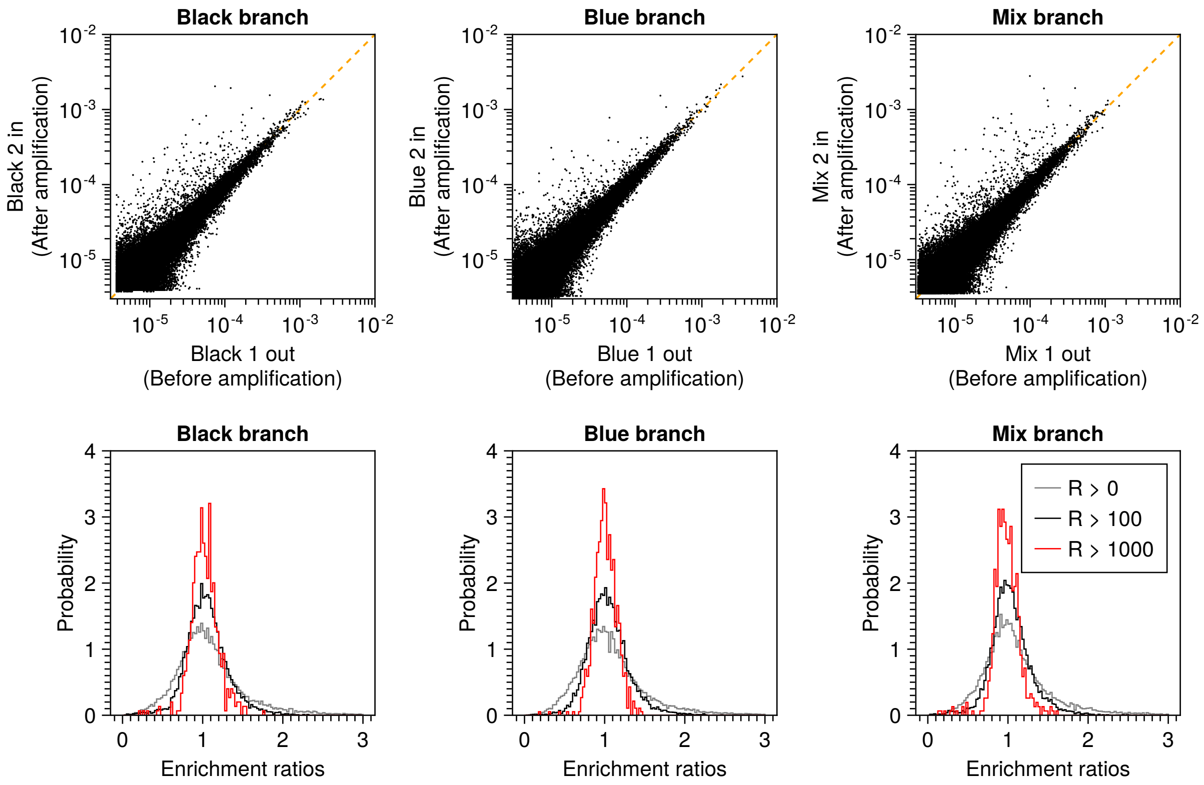

FIG. F: Sequencing reads are collected before and after amplification, after the first round of selection is finished and before starting the second round of selections. This was done for each branch of the experiment tree (see Fig. S3): Black branch, Blue branch, and Mix branch. The top panels show a scatter of the normalized counts before (x-axis) and after (y-axis) amplification. The Pearson correlation coefficients are: 0.97 (for Black branch), 0.98 (for Blue branch), and 0.97 (for Mix branch). The bottom panels show histograms of the corresponding enrichment ratios (see Eq. (S1)), after filtering out sequences with counts below a threshold (indicated in the legend). The histograms concentrate around 1, indicating no selection.

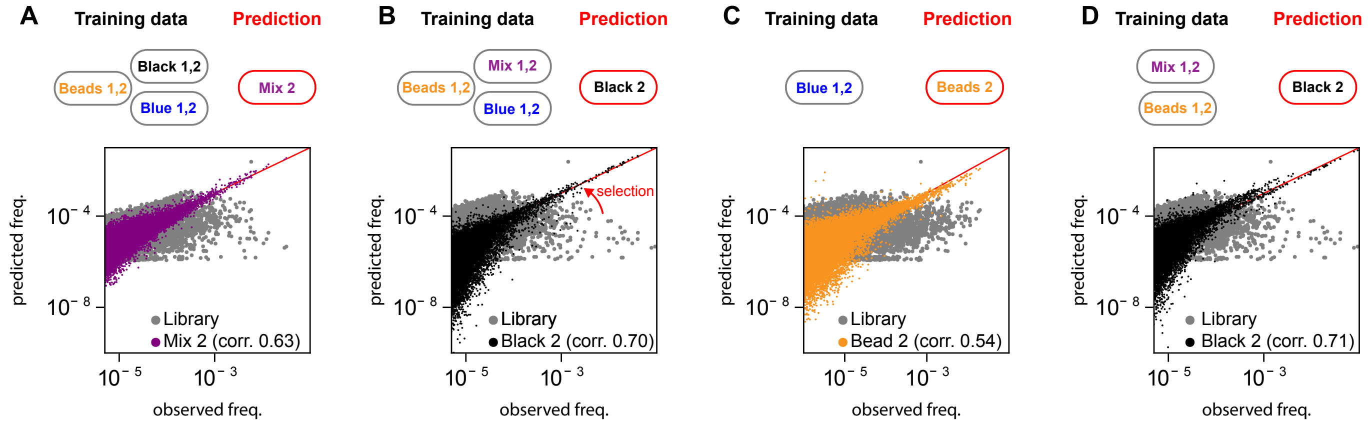

FIG. G: Same figure as Fig. 2 in the main text, employing using model with independent site as described in Eq. S14. The model predicts accurately the evolution of sequence variants abundances in response to multiple selective pressures. We considered different tasks of increasing difficulty, depending on the training set used: **A.** Model trained on the experiments with Black, Blue complexes, and empty Beads, and prediction evaluated with a mixture of the Black and Blue complexes; **B.** Model trained on experiments with a mixture of Black and Blue complexes, Blue complexes only, and naked Beads, with predictions evaluated on the experiment with Black complexes only; **C.** Model trained on experiments with Blue complexes only, and predictions evaluated on experiments with naked Beads; **D.** Model trained on experiments with a mixture of Black and Blue complexes and naked Beads, and predictions evaluated on experiments with Black complexes only. The panels show scatter plots of the observed (x-axis) vs. predicted sequence frequencies (y-axis), with the initial library abundances shown in gray for comparison. The Pearson correlation between empirical selectivities and the model-predicted selectivities for each task are given in the legend and in Table A. In all cases p-values (from Student's test) are $< 10^{-90}$.

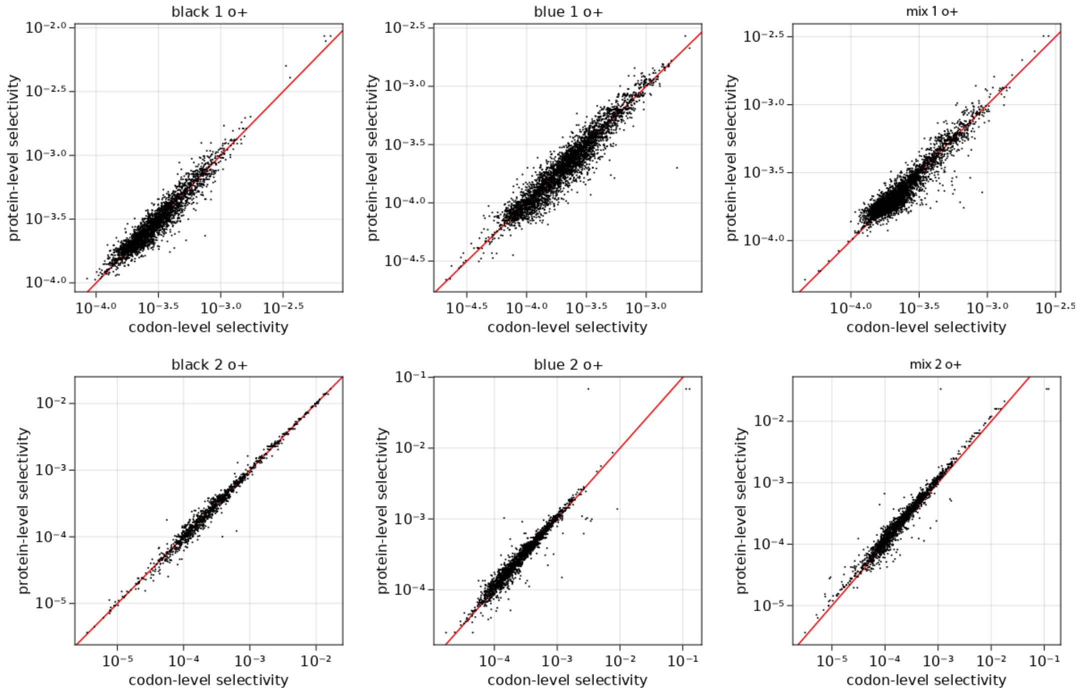

FIG. H: Each amino-acid sequence can correspond to several nucleotide variants due to codon degeneracy. The plots show a comparison of empirical enrichments (Eq. (S1)) for codon-sequences vs. the equivalent amino-acid sequences. Each panel corresponds to one experiment. Effects due to codon bias would be revealed by strong systematic dispersion in these plots. Sequences with less than 50 copies (at nucleotide or amino-acid level) are filtered out.

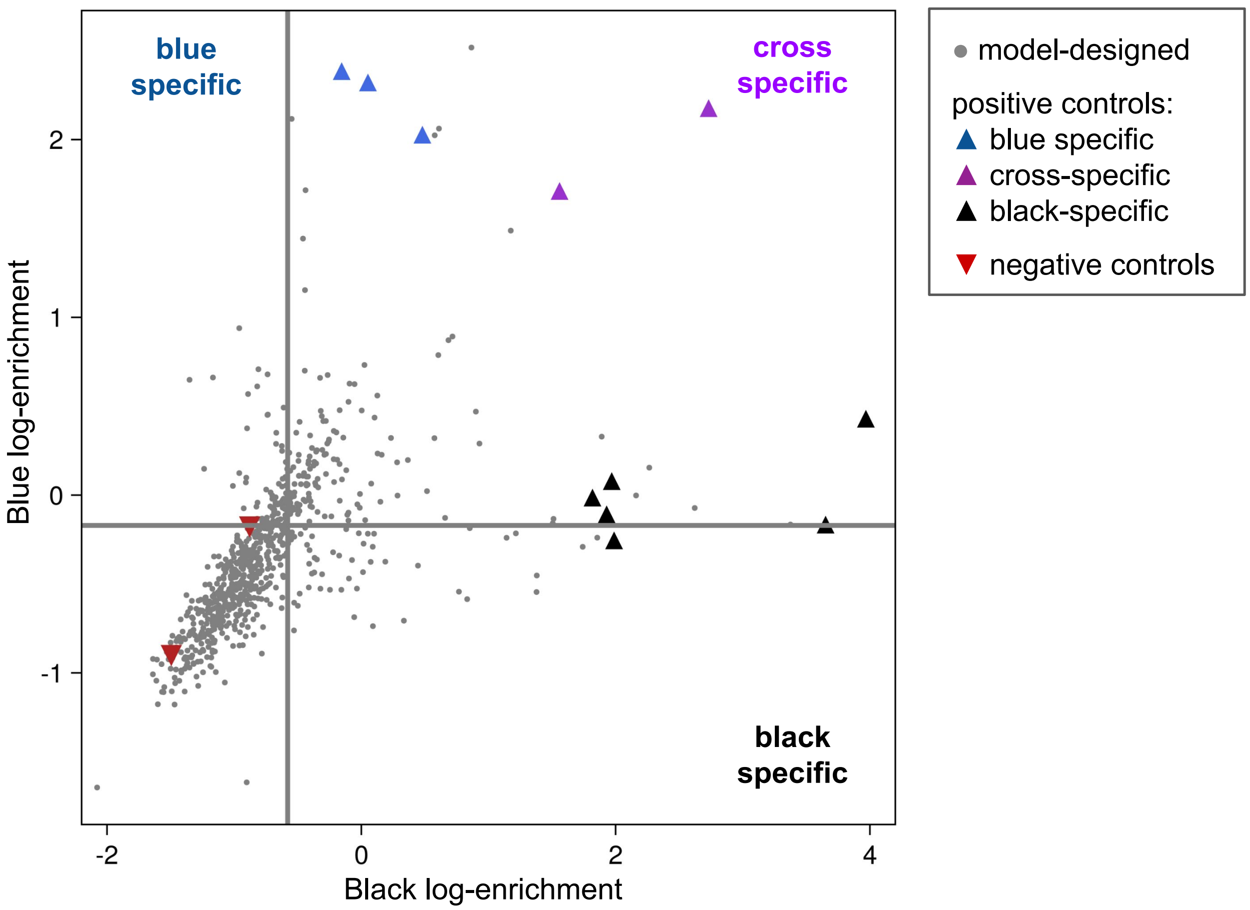

FIG. I: Experimental enrichment of model-designed sequences and controls. This figure illustrates the experimental enrichment results for model-generated sequences, marked in gray, and a selection of control sequences that serve as references and provide a sanity check for the experimental data. Among the control sequences are the best specific binders, cross-specific binders, and two negatively selected sequences based on experimental enrichments from the model-training experiments. The horizontal lines represent the two predefined thresholds used to evaluate the generative performance, as detailed in Figure 3 of the main text.

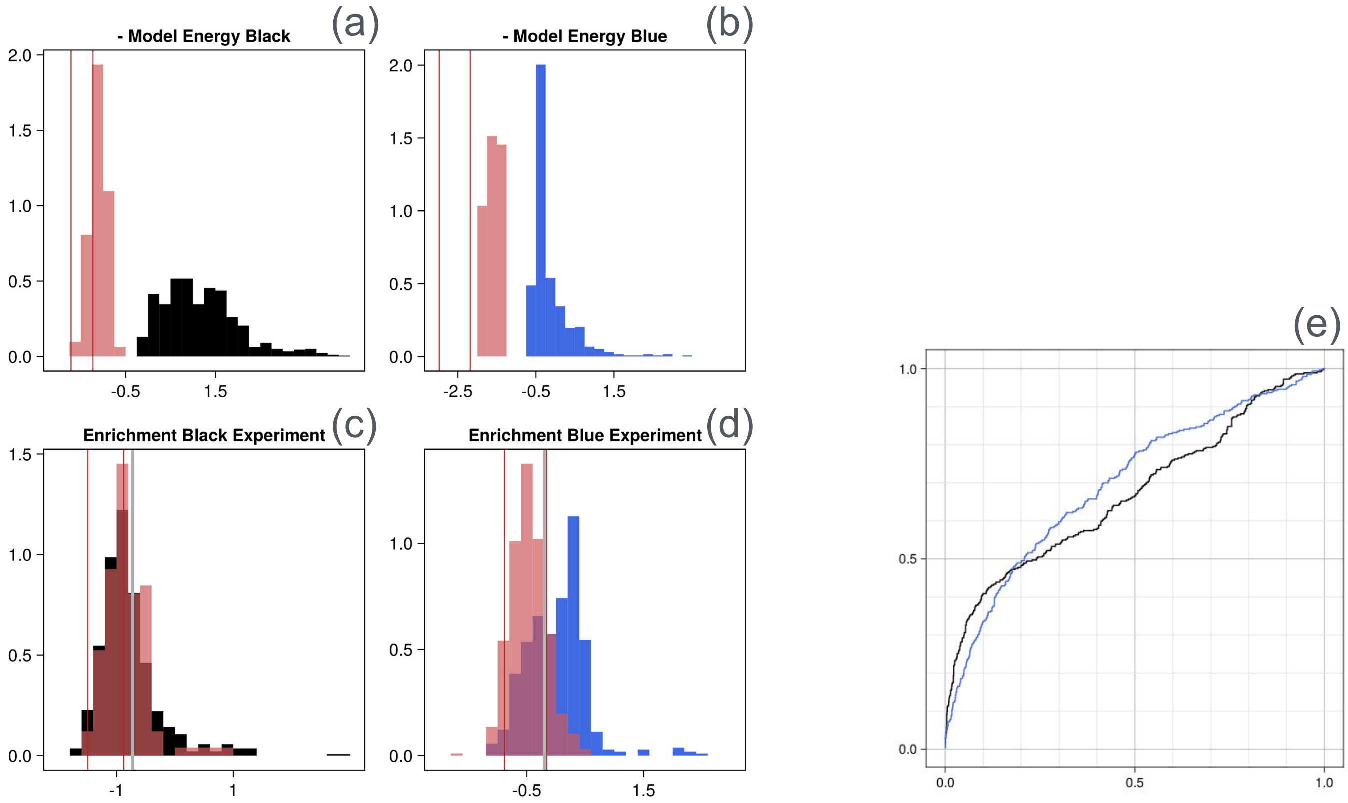

FIG. J: Validation of high-affinity antibodies generated via single-mode optimization. Designing high-affinity binders for a specific ligand, regardless of their affinity to other ligands, is a relatively easier task compared to selecting a specificity profile, as it involves no additional constraints on the optimization of a single-model energy. In panels (a) and (b), the histograms depict the energies of designed sequences. Those in black and blue represent sequences predicted to exhibit high affinity for their respective ligands, while those in red are predicted to have low affinity. Red lines correspond to negative controls (see Fig. S9). Below these panels, two histograms illustrate the enrichment of these same sequences in validation experiments. Panel (e) showcases ROC curves for binder prediction using the model. Sequences above the threshold, denoted by the gray lines in panels (c) and (d), are considered binders. This threshold is set as the average of the enrichments plus one standard deviation. The model energy ranks these sequences, and the ROC curves are computed for both the black and blue ligand experiments. The Area Under the Curve (AUC) values are 0.64 for black and 0.66 for blue.

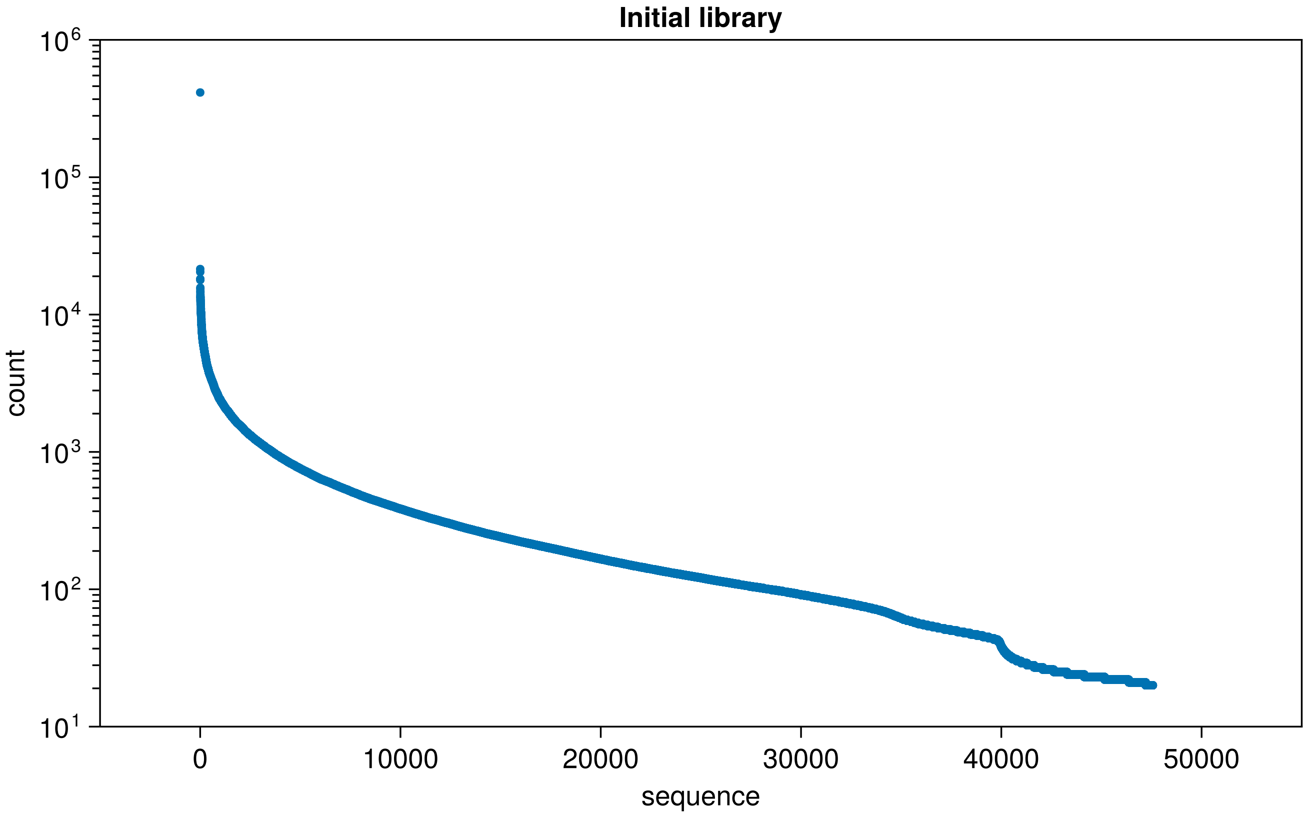

FIG. K: Number of reads of the initial library. Variants are displayed in descending order (from the most numerous variant to the less numerous one). The distribution is strongly non-uniform (note the log-scale on the y-axis).

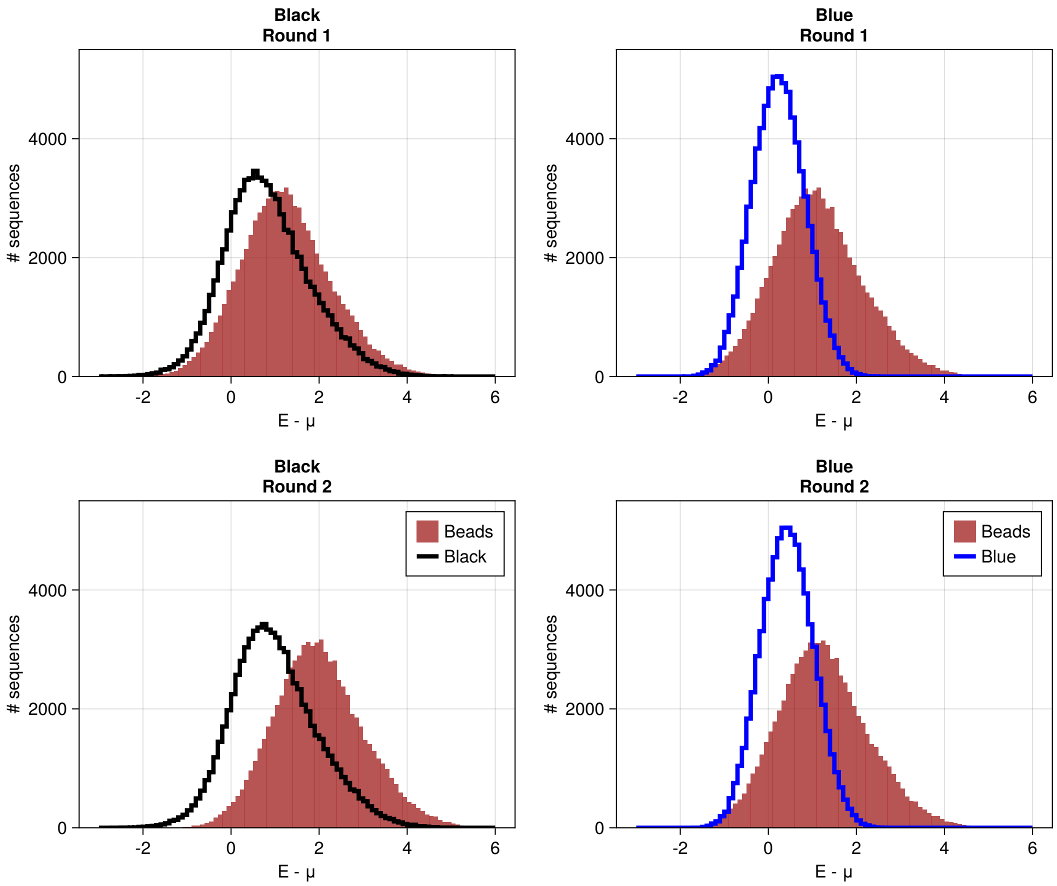

FIG. L: Histograms of $E_{sw} - \mu_{wt}$. The plots show the number of sequences s with a given value of $E_{sw} - \mu_{wt}$. The first column corresponds to experiments carried out with the Black target, and w is either the Black-bound mode (black line) or the Beads-bound mode (in brown). The right column corresponds to experiments carried out with the Blue target, and w is either the Blue-bound mode blue line) or the Beads-bound mode (in brown). The first and second rows correspond to the first and second rounds of selection. The binding energies to the Black and Blue complexes are generally lower than the Beads binding energies.

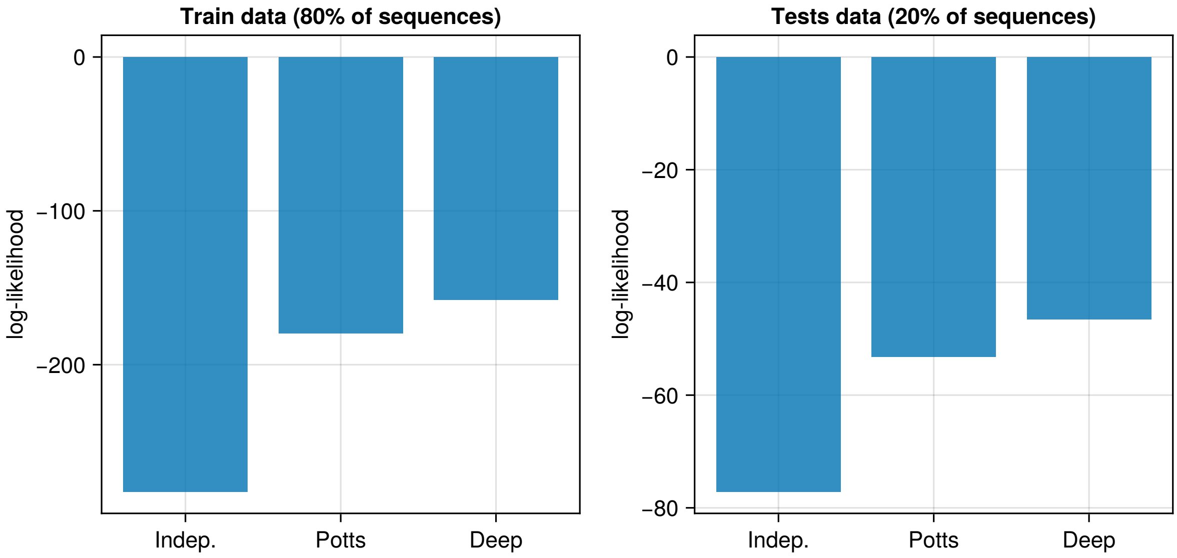

FIG. M: Comparison of models for the sequence-to-energy mapping. We considered three models for the sequence to function mapping: **Indep.**, where the energy assigned to each sequence variant in each thermodynamic state is computed under an independent-site assumption (this model considers only qL site parameters, for each state); **Potts**, where in addition to the site parameters, the model includes $L(L-1)/2 \times q^2$ pairwise couplings between all pairs of sites; and **Deep**, where the energy is computed using the neural network (NN) architecture we have presented above. We then conducted a training/validation test to demonstrate the advantage of our NN architecture. These models were trained on a randomly selected subset of 80% of the sequences in the experimental data. After training converged, we computed the likelihood (the closer to zero the better) of the observed sequence counts, both in the training split, and in the remaining 20% of sequences in a withheld validation split. The results are shown in the barplot figure.

Target	# generated in region	# generated outside	# others in region	# others outside	p-value
Black	39	441	49	975	**0.008**
Blue	23	101	107	1041	**0.002**
cross	71	88	605	1240	**0.001**

TABLE C: Regarding the data in Figure 3, p-values were computed using the Fisher exact test. We considered the null hypothesis that the generated antibodies, specific for Black, Blue, or cross-specific, are drawn randomly. More precisely, for each of these three tasks, we considered two groups of antibodies: those designed for the task and all the others. For both groups, we computed the number of times they are in the right region of the enrichment plane or outside (see Figure 3). We then assessed the probability that the occurrences of the designed antibodies are drawn from the same distribution as the others. The table reports these numbers and the corresponding p-values.

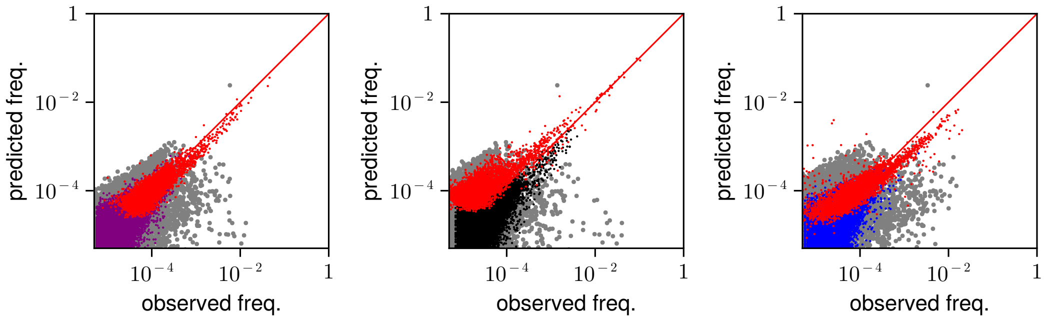

FIG. N: The model is trained on the 95% set of sequences with the lowest counts over the entire experiment. Then we compute the predicted frequencies in the final round for the remaining 5% of sequences. The graph compares this prediction with the actual frequencies. Points in red correspond to the 5% sequences with the highest overall abundances. Other colors follow the same convention as in Fig. 2 of the main text. The panels correspond to the last selection rounds of the experiments with both targets (left panel), black target (middle panel), and blue target (right panel). The Pearson correlations (test set only) are: Both: 0.86; Black: 0.89; Blue: 0.84.
